# Targeting the LHX1-LDB1 Complex Restores STING-dependent Senescence Surveillance and Inhibits Head and Neck Cancer Progression

**DOI:** 10.7150/ijbs.123790

**Published:** 2026-01-01

**Authors:** Mingshu Long, Yang Chen, Ruixue Du, Jiejie Yang, Jingjing Wang, Yunqing Sun, Xuezhang Tian, Shaowei Wang, Yunhong Zhong, Weilian Liang, Junjie Zhang, Zhengjun Shang

**Affiliations:** 1 State Key Laboratory of Oral & Maxillofacial Reconstruction and Regeneration, Key Laboratory of Oral Biomedicine Ministry of Education, Hubei Key Laboratory of Stomatology, School & Hospital of Stomatology, Wuhan University, Wuhan, China.; 2 Department of Oral and Maxillofacial-Head and Neck Oncology, School & Hospital of Stomatology, Wuhan University, Wuhan, China.; 3 Department of Oral and Maxillofacial Surgery, School & Hospital of Stomatology, Wuhan University, Wuhan, China.; 4 Frontier Science Center for Immunology and Metabolism, Medical Research Institute, Wuhan University, Wuhan, China.; 5 Day Surgery Center, School and Hospital of Stomatology, Wuhan University, Wuhan, China.; 6 Taikang Center for Life and Medical Sciences of Wuhan University, Wuhan, China.

**Keywords:** LHX1, STING signaling, SASP, cancer stem cells, peptide therapeutics

## Abstract

The senescence-associated secretory phenotype (SASP) exerts dual roles in tumor suppression and progression, yet how it is regulated in head and neck squamous cell carcinoma (HNSCC) remains unclear. Here, we identify LIM homeobox 1 (LHX1) as a key transcriptional suppressor of STING, whose downregulation enables evasion of SASP-mediated tumor surveillance. Notably, high LHX1 expression correlated with poor prognosis in HNSCC patients. Mechanistically, LHX1, in complex with LDB1, directly bound to the *STING* promoter to mediate transcriptional repression via the deposition of the repressive histone mark H3K9me3, thereby blocking SASP activation. Depletion of *LHX1* restored STING-dependent SASP and impaired cancer stem cell self-renewal. Therapeutic disruption of the LHX1-LDB1 complex using engineered peptides re-activated STING signaling, induced SASP, and significantly suppressed tumor growth. In this study, we employed human and mouse-derived HNSCC cell lines, xenograft models, and clinical samples to assess the functional relevance of LHX1 in regulating SASP and tumor progression. Our findings reveal LHX1 as a master transcriptional repressor of STING-mediated senescence and highlight the therapeutic potential of targeting the LHX1-LDB1 axis to restore tumor-suppressive SASP in HNSCC.

## Introduction

Cell senescence was first described in 1961 as a mechanism explaining the limited proliferative capacity of fibroblasts^1^. Senescence is a complex biological phenomenon that involves crucial alterations in cell morphology and physiology, with cell cycle arrest being the most prominent feature^2^,^3^. Moreover, senescent cells adopt a senescence-associated secretory phenotype (SASP), secreting pro-inflammatory cytokines, growth factors, and proteases to remodel the microenvironment via autocrine/paracrine signaling^4^. Interestingly, SASP factors can induce senescence in surrounding cells, thereby promoting the spread and accumulation of senescent cells^5^. Compelling evidence has established that cellular senescence and the associated SASP exert a dual, context-dependent influence on cancer development^6^,^7^. On one hand, acute SASP activation can trigger immune surveillance and suppress tumor progression, as evidenced by studies showing that the ablation of senescent cells inhibits tumorigenesis and cancer-related death in mice^8^,^9^. Besides, many cancer therapies, including radiotherapy, chemotherapy, and certain targeted agents, restrict tumor growth through therapy-induced senescence^10^. On the other hand, chronic SASP signaling paradoxically fosters a tumor-promoting microenvironment by facilitating angiogenesis, extracellular matrix remodeling, and cancer stem cell (CSC) reprogramming^11^,^12^. Stimulator of interferon genes (STING) is a key regulator of SASP induction in response to cytoplasmic chromatin fragments^13^. Given the potential detrimental effects of a persistent SASP, it is perhaps unsurprising that the STING pathway is frequently subject to epigenetic silencing in various cancers^14^, representing a potential immune evasion mechanism. However, how STING-dependent SASP is specifically regulated in different tumor contexts, such as HNSCC, and whether its upstream regulators can be therapeutically targeted, remains elusive.

Head and neck cancers rank as the sixth most prevalent malignancy globally, with approximately 946 000 new cases and 482 000 deaths reported in 2022, and over 90% of these malignancies are classified as head and neck squamous cell carcinoma (HNSCC)^15^. Current therapeutic strategies combining surgery, radiotherapy, chemotherapy, and targeted immunotherapy achieve only a five-year survival rate of 50%, largely due to tumor heterogeneity^16^-^18^. CSC, a self-renewing subpopulation capable of multi-lineage differentiation, are key drivers of this heterogeneity, contributing to tumor recurrence, metastasis, and therapy resistance^19^-^21^. Accumulating evidence highlights the role of CSC in HNSCC progression^22^-^25^. While our prior work revealed that CSC in HNSCC leverages heightened autophagy for survival under stress^26^, the molecular mechanisms underlying their escape from senescence and maintenance of self-renewal capacity remain elusive. Targeting these pathways may unlock novel therapies against HNSCC.

LIM homeobox 1 (LHX1), a LIM-HD transcription factor essential for embryonic organogenesis, contains two tandemly repeated cysteine-rich LIM domains upstream of the homeodomain^27^. The homeodomain functions as a DNA-binding domain, directly interacting with DNA, while the LIM domains facilitate LHX1 to form transcriptional complexes with various protein partners, which is a prerequisite for LHX1 to target specific genes for transcriptional regulation^28^,^29^. Emerging evidence has identified LHX1 as an oncogene^30^-^35^. For instance, Dormoy *et al.* demonstrated that LHX1 accelerates renal cell carcinoma growth, partly through the activation of the PI3K/AKT and NF-κB signaling pathways^34^. In uterine corpus endometrial carcinoma, LHX1 has been identified as both a diagnostic and prognostic biomarker, with its expression positively correlating with epithelial-mesenchymal transition (EMT) status^35^. Notably, bioinformatic analyses by Wang *et al.* specifically identified LHX1 as being highly expressed in tongue squamous cell carcinoma, a major subtype of HNSCC, and its elevated level is a marker of unfavorable patient outcomes^36^. Despite these advances, the roles of LHX1 in HNSCC and CSC biology remain unexplored.

In this study, we reveal that LHX1 is upregulated in HNSCC and predicts poor prognosis. We identify a previously unrecognized mechanism wherein LHX1 suppresses STING-driven SASP, facilitating CSC maintenance and tumor progression. Mechanistically, LHX1 interacts with LDB1 to repress *STING* transcription, and disrupting this interaction abolishes LHX1's ability to sustain CSC properties. Finally, we propose a peptide-based strategy to inhibit the LHX1-LDB1 interaction, restoring STING-SASP signaling and impairing tumor progression. These findings establish LHX1-mediated senescence evasion as a CSC vulnerability and provide a new therapeutic avenue for HNSCC.

## Materials and Methods

### Clinical samples

In this study, 21 clinical HNSCC samples and corresponding adjacent normal tissues were collected from the School and Hospital of Stomatology, Wuhan University, for LHX1 protein and mRNA expression detection analysis. Written informed consent was obtained from all participants. The study design conformed to the principles of the 1975 Declaration of Helsinki and was approved by the Ethics Committee of School and Hospital of Stomatology, Wuhan University (approval number: WDKQ2024A11). Detailed patient information is provided in Supplementary Table S1.

### Cell culture

The CAL27, SCC25, and HEK293T cell lines were purchased from ATCC, while FaDu and HaCaT cell lines were obtained from National Collection of Authenticated Cell Cultures. The HN4 cell line was kindly provided by School of Dentistry, University of Maryland. CAL27, HaCaT, HN4, and HEK293T cells were cultured in DMEM (Gibco, NY, USA, Cat. #11965092), SCC25 cells in DMEM/F12 (Gibco, Cat. #11320033), and FaDu cells in MEM (Gibco, Cat. #11095080), supplemented with 10% FBS (Lonsera, Shanghai, China, Cat. #S711-001). HIOEC cell line was kindly provided by Dr. Chengzhang Li (Wuhan University) and cultured in KGM^TM^-2 BulletKit (Lonza, Basel, Switzerland, Cat. #CC-3103 and #CC-4152). All cells were cultured at 37°C and 5% CO_2_ and passaged fewer than 15 times during the experiment. *Mycoplasma* contamination was checked bi-weekly.

### Tissue microarray and Immunohistochemical (IHC) staining

The tissue microarray was constructed as described^37^. IHC staining for LHX1 was performed as follows. Tissue slides were deparaffinized in xylene and rehydrated through a graded ethanol series. Antigen retrieval was conducted using citrate buffer. Endogenous peroxidase activity was blocked with hydrogen peroxide, followed by incubation to block non-specific binding sites. The slides were then incubated with the primary LHX1 antibody (Abclonal, Wuhan, China, Cat. #A17344) and the HRP-labeled ` secondary antibody (MXB, Fuzhou, China, Cat. #KIT-5001). Staining was developed using 3,3'-diaminobenzidine (MXB, Cat. #DAB-0031). Finally, the stained slides were scanned and the staining intensity was quantified using Case Viewer (Version 2.4). The primary antibodies used for IHC staining of harvested tumor tissues included Ki67 (Proteintech, Chicago, USA, Cat. #28074-1-AP, RRID: AB_2918145), Cleaved Caspase-3 (Proteintech, Cat. #25128-1-AP, RRID: AB_3073913), STING (Proteintech, Cat. #19851-1-AP, RRID: AB_10665370), P-TBK1/NAK-S172(Abclonal, Cat. #AP0847, RRID: AB_2771611), and P-IRF3 (Ser396) (Proteintech, Cat. #29528-1-AP, RRID AB_2935415).

### The Cancer Genome Atlas (TCGA) and Gene Expression Omnibus (GEO) analysis

LHX1 expression and its correlation with the pathological grade, clinical stage, age, and gender of HNSCC patients in TCGA database were analyzed using Sangerbox 3.0 (http://sangerbox.com). RNA sequencing (RNA-seq) data of GSE178537 (16 paired HNSCC and adjacent normal tissue samples) and GSE202048 (11 paired HNSCC and adjacent normal tissue samples) were downloaded from the GEO database. LHX1 expression levels were extracted from these datasets. To evaluate the differences in LHX1 expression between cancerous and adjacent normal tissues, a paired t-test was performed using GraphPad Prism software (version 9.3.1).

### Transcriptome profiling

Ctrl and *LHX1*-knockout FaDu cells were sent to NovelBio (Shanghai, China) for RNA-seq. Briefly, RNA was extracted from the cells, and cDNA libraries were constructed. Library quality was assessed using the Agilent 2200 system, and sequencing was performed on a DNBSEQ-T7 platform with a 150-bp paired-end run. Differentially expressed genes (DEGs) were identified using the DESeq2 algorithm with the following criteria: Fold Change > 2, adj* P*-value < 0.05. Gene Ontology (GO), Kyoto Encyclopedia of Genes and Genomes (KEGG), and Gene Set Enrichment Analysis (GSEA) were performed to investigate the biological significance of the identified DEGs.

### Constructs

sgRNAs targeting *LHX1*, *STING*, and *LDB1* were cloned into the Lenti-CRISPRv2-Puro/Blast vector. The corresponding primers for sgRNAs are listed in Supplementary Table S2. The pLV3-CMV-FLAG-LHX1-Puro and pGL4-STING-Promoter-Fluc plasmids were obtained from Miaoling (Wuhan, China, Cat. #P63334 and #P76365). LDB1 was amplified from FaDu cells cDNA and subsequently subcloned into the pLV3-CMV-HA-Puro vector. For the rescue assay, LHX1 mutants [1m (C28G), 2m (C88G), 3m (C28G, C88G), ΔLIM (Δ3-117), and ΔHOX (Δ180-242)] were generated by site-directed mutagenesis and cloned into the PCDH-CMV-FLAG-NEO vector. The LDB1 mutant ΔLID (Δ336-375) was similarly generated and subcloned into the PCDH-CMV-HA-Hygro vector. For protein purification, the LHX1-LIM domains (3-117) were subcloned into pGEX6P1-GST vector, and the LDB1 was subcloned into pET28a-His vector.

### Stable cell line generation

psPAX2, VSVG, and the lentiviral plasmids were co-transfected into HEK293T cells using standard transfection protocols. 48h post-transfection, viral supernatants were collected and used to infect cells. After 6h of incubation, the medium was replaced with fresh culture medium. Cells were then selected with the appropriate antibiotics 48h post-transduction. Following 72h of drug selection, knockout or overexpression efficiency was assessed.

### Western blot and RT-qPCR

For Western blot, tissues or cells were lysed in RIPA buffer (Biosharp, Anhui, China, Cat. #BL2349A). Total proteins were separated by SDS-PAGE, then transferred onto a PVDF membrane. The membrane was blocked and incubated with primary and secondary antibodies. Protein bands were detected using the Odyssey system (LI-COR, Nebraska, USA, Cat. #3350-00) and quantified by Image Studio (Version 5.2.5). Primary antibodies used in this study included LHX1 (Abclonal, Cat. #A17344, RRID: AB_2770177), β-Actin (Proteintech, Chicago, USA, Cat. #66009-1-Ig and #20536-1-AP, RRID: AB_2687938 and AB_10700003), pRb (Ser807/811) (CST, Cat. #9308, RRID: AB_331472), p16 (Proteintech, Cat. #10883-1-AP, RRID: AB_2078303), p21(Proteintech, Cat. #10355-1-AP, RRID: AB_2077682), STING (Proteintech, Cat. #19851-1-AP, RRID: AB_10665370), TBK1 (Proteintech, Cat. #28397-1-AP, RRID AB_2881132), P-TBK1/NAK (Ser172) (CST, Cat. #5483T, RRID:AB_10693472), IRF3 (Proteintech, Cat. #11312-1-AP, RRID AB_2127004), P-IRF3 (Ser396) (Proteintech, Cat. #29528-1-AP, RRID AB_2935415), FLAG (Proteintech, Cat. #20543-1-AP and #66008-4-Ig, RRID: AB_11232216 and AB_2918475), HA (Proteintech, Cat. #51064-2-AP and #66006-2-Ig, RRID: AB_11042321 and AB_2881490), and LDB1(Proteintech, Cat. #68721-1-Ig, RRID: AB_3670413). Secondary antibodies used were IRDye 800CW (Licorbio, Cat. #926-32210 and #926-32211, RRID: AB_2687825 and AB_621843) and IRDye 680RD (Licorbio, Cat. #926-68070 and #926-68071, RRID: AB_10956588 and AB_10956166).

For RT-qPCR, total RNA was extracted from tissues or cells using TRIzol (Takara, Osaka, Japan, Cat. #9108). cDNA was synthesized using the HiScriptⅢ 1st strand cDNA synthesis Kit (Vazyme, Nanjing, China, Cat. #R312-01). RT-qPCR was performed using the Bio-Rad CFX Connect with SYBR Green PCR Master Mix (Bimake, Cat. #B21403). The expression levels of the target genes were calculated using the ΔΔCt method, with *ACTB* as the reference gene. The primers used for RT-qPCR are listed in Supplementary Table S3.

### Colony formation and sphere formation assays

For the colony formation assay, 200 cells were inoculated into each well of a 12-well plate. After two weeks, the cells were fixed with paraformaldehyde and stained with crystal violet. Colonies consisting of 50 or more cells were counted and included in the analysis. For sphere formation assay, 1,000 cells were seeded into each well of a 6-well low-attachment plate (Corning, NY, USA, Cat. #3471). Cells were cultured for 10 days in DMEM/F12 medium supplemented with B-27 (Gibco, Cat. #17504044), EGF (PeproTech, New Jersey, USA, Cat. #AF-100-15), FGF (PeproTech, Cat. #100-18B), and Sodium heparin (MCE, New Jersey, USA, Cat. #9041-08-1). Spheres with a diameter of 75 μm or greater were counted and statistically analyzed.

### Enzyme-Linked Immunosorbent Assay (ELISA)

The levels of IL-1α, IL-6, IL-8, and cGAMP in the conditioned media were measured using ELISA kits following the manufacturer's instructions. ELISA kits used in this study included IL-1α (Proteintech, Chicago, USA, Cat. #KE00877), IL-6 (Proteintech, Cat. #KE00139), IL-8 (Proteintech, Cat. #KE00275), cGAMP (Cayman Chemical, Ann Arbor, USA, Cat. #501700).

### Senescence-associated β-galactosidase (SA-β-gal) Staining

Cellular senescence was assessed by SA-β-gal staining according to the protocol provided by the Senescence β-galactosidase Staining Kit (Beyotime, Shanghai, China, C0602). Cells were fixed, washed, and incubated with the X-Gal-containing staining solution overnight at 37 °C in a CO₂-free incubator. Following staining, cells were rinsed and imaged. Senescent cells, identified by blue cytoplasmic staining, were quantified from at least three random fields per sample.

### Conditioned medium transfer assays

For conditioned medium (CM) collection, *LHX1*-knockout cells and their control counterparts were cultured in serum-free medium. The CM was harvested after 48 hours, followed by centrifugation at 1,500 rpm for 10 minutes to remove cellular debris. The supernatant was collected, aliquoted, and either used immediately for subsequent treatments or stored at -80°C. For CM treatment, recipient cells were incubated with a 1:1 mixture of fresh medium and the collected CM for the indicated durations.

### Luciferase assay

HEK293T cells were cultured on a 24-well plate and transfected with 50 ng pGL4-STING-Promoter-Fluc plasmid, 20 ng phRL-TK plasmid, 100 ng pLV3-CMV-FLAG-LHX1 plasmid. 24h post-transfection, luciferase activity was measured using the Dual-Luciferase Reporter Assay Kit (Beyotime, Shanghai, China, Cat. #RG027). *STING* transcriptional activity was determined by the ratio of firefly luciferase to Renilla luciferase activity.

### ChIP assay

ChIP was performed following the manufacturer's instructions for the Sonication ChIP Kit (Abclonal, Cat. #RK20258). Briefly, 1×10^7^ cells were collected and crosslinked, followed by nuclear extraction and chromatin sonication. Chromatin fragmentation and concentration were assessed, and immunoprecipitation was carried out. The chromatin was then eluted, de-crosslinked, and DNA was purified using a centrifugal column. ChIP results were analyzed by PCR and qPCR. The primers used in the ChIP assay are provided in Supplementary Table S4.

### Immunoprecipitation-mass spectrometry (IP-MS)

1×10^7^ FaDu cells were lysed in 1 mL NP-40 buffer (Biosharp, Cat. #BL653A). The lysate was incubated with 2 µg of LHX1 antibody or IgG at 4 °C for 4h. Then, protein A agarose (MCE, Cat. #HY-K0213) was added, and the mixture was incubated overnight at 4°C. The antigen-antibody complexes were then sent to SpecAlly Life Technology Co., Ltd (Wuhan, China) for IP-MS analysis.

### Co-immunoprecipitation (CoIP) assay

HEK293T, CAL27, and FaDu cells were lysed in NP-40 buffer. The lysates were then incubated with the corresponding primary antibodies or IgG at 4 °C for 4h. Protein A agarose was then added, and the mixture was incubated overnight at 4°C to capture the antigen-antibody complexes. After centrifugation to collect the precipitate, the samples were resuspended in 30 μL of protein loading buffer, heated at 95 °C for 10 minutes, and subsequently subjected to Western blot analysis.

### GST-pulldown assay

The pGEX6P1-GST, pGEX6P1-GST-LHX1-LIMs (3-117), and pET28a-His-LDB1 were expressed in *E. coli* BL21 cells and induced with IPTG for 18h at 25 ℃. Following induction, bacterial cells were disrupted using ultrasonic treatment. The supernatants containing His-LDB1, GST, and GST-LHX1-LIMs were collected and incubated with Ni-NTA resin (MCE, Cat. #HY-K0210) and Glutathione Agarose (MCE, Cat. #HY-K0211), respectively. After washing with the appropriate buffer, the bound proteins were eluted using an elution buffer. The eluted proteins were subsequently used for GST-pulldown assays.

GST and GST-LHX1-LIMs were incubated with His-LDB1 protein in binding buffer and mixed with Glutathione Agarose. The mixture was incubated with rotation for 4h at 4 °C. After centrifugation, the supernatant was discarded, and the bound proteins were resuspended in protein loading buffer. The samples were boiled at 95 °C for 10 minutes, separated by SDS-PAGE, and then stained with Coomassie Brilliant Blue.

### Peptides

The TAT, TAT-AE, TAT-VM, TAT-DRI, TAT-AE-DRI, and TAT-VM-DRI peptides were synthesized by QYAOBIO (shanghai, China). The GFP, TAT-GFP, TAT-AE-GFP, and TAT-VM-GFP proteins were expressed using the pGEX-6P-1 vector.

### Immunofluorescence (IF)

Cells were plated onto coverslips. After cell adhesion, they were sequentially fixed, permeabilized, and blocked. The cells were then incubated overnight at 4 °C with primary antibodies targeting LHX1 (Abclonal, Cat. #A17344, rabbit polyclonal) and LDB1 (Proteintech, Cat. #68721-1-Ig, mouse monoclonal). After washing with PBS, the cells were incubated with secondary antibodies, 488-conjugated Goat anti-Rabbit (Abclonal, Cat. #AS073) and 594-conjugated Goat anti-Mouse (Abclonal, Cat. #AS054), at room temperature for 30 minutes. The samples were mounted using a DAPI-containing mounting medium. Immunofluorescence images were acquired using a confocal microscope (Zeiss LSM880).

### Animal studies

For the *in vivo* extreme limiting dilution assay (ELDA), 1×10^3^, 1×10^4^, 1×10^5^ tumor cells were subcutaneously injected into the right flank of BALB/c nude mice. 30 days post-inoculation, tumor numbers were counted, and tumor-initiating frequency (TIF) was calculated by the ELDA software^38^.

To assess the therapeutic effects of the peptides on HNSCC, both subcutaneous and orthotopic xenograft tumor models were used. For subcutaneous models, 3×10^6^ CAL27 and FaDu cells were injected into the flanks of BALB/c nude mice. For orthotopic models, 3×10^5^ MOC1 and MOC2 cells were inoculated into the tongues of C57BL/6J mice. Mice were treated with intraperitoneal injections of 2 µmol/kg TAT-DRI or 1 µmol/kg TAT-AE-DRI and 1 µmol/kg TAT-VM-DRI every 3 days. Tumor size and mouse survival rates were monitored and recorded throughout the study. At the end of the experiment, animals were euthanized using an overdose of carbon dioxide. The study was approved by the Ethics Committee of School and Hospital of Stomatology, Wuhan University (approval number: S07924110A).

### Statistical analysis

All experiments were conducted in triplicate. Statistical analyses were performed using GraphPad Prism software (version 9.3.1). The correlation between LHX1 expression and clinical stage, pathological grade, age, and gender was assessed using the Pearson *χ2* test and the *t* test. Survival data were analyzed using Kaplan-Meier survival curves and Log-rank test. For comparisons between two groups, the paired or unpaired *t* test was used, while one-way ANOVA was employed for comparisons across multiple groups. Significant differences were indicated as follows: *P < 0.05; **P < 0.01; ***P < 0.001; ****P < 0.0001; ns, not significant.

## Results

### LHX1 is upregulated in HNSCC and predicts unfavorable prognosis

**Figure 1:**
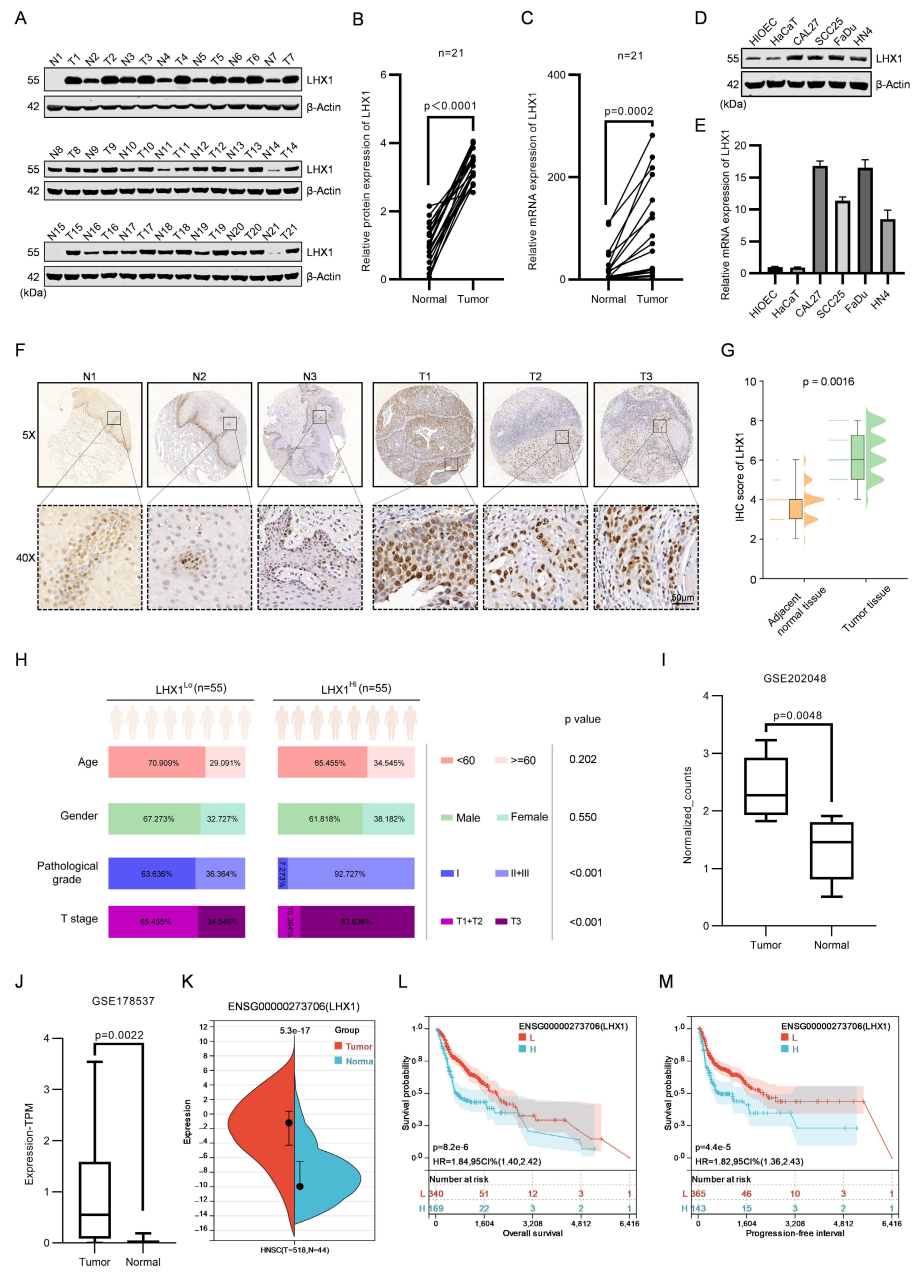
** LHX1 is upregulated in HNSCC and predicts unfavorable prognosis. A,** Immunoblotting of LHX1 in 21 paired HNSCC and adjacent normal tissues. **B,** Statistical analysis of the relative LHX1 protein levels. *n* = 21, paired *t* test.** C,** RT-qPCR analysis of *LHX1* in 21 paired HNSCC and adjacent normal tissues. *n* = 21, paired *t* test. **D,** Immunoblotting of LHX1 in HNSCC cells (CAL27, SCC25, FaDu, and HN4) and normal epithelial cells (HIOEC and HaCaT). **E,** RT-qPCR analysis of *LHX1* in HNSCC cells and normal epithelial cells.** F,** IHC staining of LHX1 in tissue microarrays containing cores from 110 HNSCC tumors and 100 normal tissues. Scale bar, 50 μm. **G,** Statistical analysis of IHC staining intensity of LHX1. Data are derived from* n* = 110 independent tumor samples and *n* = 100 independent normal tissue samples, unpaired *t* test. **H,** Correlation between LHX1 expression and clinical stage, pathological grade, age, and gender in HNSCC patients. *n* = 55 for LHX1^high^, *n* = 55 for LHX1^low^, *χ2* test and unpaired *t* test. **I,** Expression of LHX1 in HNSCC and normal tissues from the GSE202048 database.* n* = 11, paired *t* test.** J,** Expression of LHX1 in HNSCC and normal tissues from the GSE178537 database.* n* = 16, paired *t* test. **K,** Expression of LHX1 in HNSCC and normal tissues from the TCGA database. *n* = 518 for tumors, *n* = 44 for normal tissues, unpaired *t* test. **L,** Correlation between LHX1 expression and OS in HNSCC patients. **M,** Correlation between LHX1 expression and PFI in HNSCC patients.

LHX1 plays essential regulatory roles in lineage differentiation and morphogenetic tissue movement during embryonic head development^29^. However, the expression and function of LHX1 in HNSCC remain largely unexplored, despite several studies suggesting its potential involvement in the progression of other cancers^30^-^35^.In this study, we analyzed the protein and RNA expression of LHX1 in 21 paired HNSCC tissues and adjacent normal tissues. The results demonstrated a statistically significant upregulation of LHX1 in neoplastic tissues (Fig. 1A-C). Moreover, LHX1 expression was notably higher in four HNSCC cell lines (CAL27, SCC25, FaDu, and HN4) compared to normal epithelial cell lines (HIOEC and HaCaT) (Fig. 1D, E). To validate these findings, IHC staining for LHX1 was performed on tissue microarrays consisting of 110 HNSCC tumors and 100 normal tissues. This analysis confirmed a marked increase of LHX1 expression in HNSCC tissues relative to adjacent normal tissues (Fig. 1F, G). Additionally, LHX1 expression was significantly associated with pathological grade and clinical stage among HNSCC patients, but not with age or gender (Fig. 1H). Further analysis of publicly available datasets (GSE202048, GSE178537, and TCGA) also revealed elevated LHX1 expression in HNSCC tissues compared to normal tissues (Fig. 1I-K). And high LHX1 expression was associated with poorer clinical outcomes, including decreased overall survival (OS), progression-free interval (PFI), disease-free interval (DFI), and disease-specific survival (DSS) rates (Fig. 1L, M; Supplementary Fig. S1A, B). Furthermore, LHX1 expression was correlated with lymph node metastasis, distant metastasis, pathological grade, and patient gender. In contrast, no significant association was found between LHX1 expression and primary tumor size, clinical stage, or patient age (Supplementary Fig. S1C-I). Taken together, these findings suggest that LHX1 is significantly upregulated in HNSCC and could serve as a prognostic biomarker for poor clinical outcomes in HNSCC patients.

### LHX1 contributes to CSC properties

**Figure 2:**
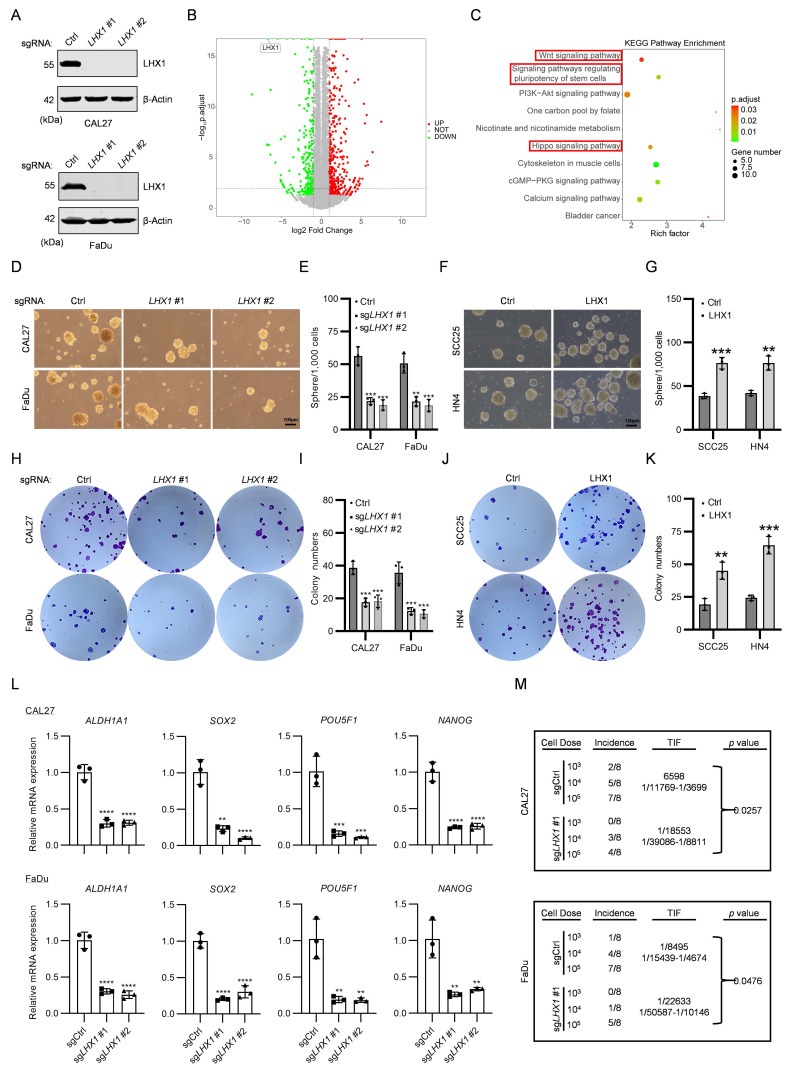
** LHX1 contributes to CSC properties. A,** Immunoblotting of LHX1 following *LHX1* knockout. **B,** Volcano plot of DEGs from RNA-seq following *LHX1* knockout in FaDu cells. **C,** KEGG analysis of 386 downregulated DEGs following *LHX1* knockout. **D,** Sphere formation assay following *LHX1* knockout. Scale bar, 100 μm.** E,** Quantification of sphere numbers following *LHX1* knockout. *n* = 3, one-way ANOVA. **F,** Sphere formation assay following LHX1 overexpression. Scale bar, 100 μm. **G,** Quantification of sphere numbers following LHX1 overexpression.* n* = 3, unpaired *t* test. **H,** Colony formation assay following *LHX1* knockout. **I,** Quantification of colony numbers following *LHX1* knockout. *n* = 3, one-way ANOVA.** J,** Colony formation assay following LHX1 overexpression. **K,** Quantification of colony numbers following LHX1 overexpression. *n* = 3, unpaired *t* test. **L,** RT-qPCR analysis of A*LDH1A1, SOX2, POU5F1,* and *NANOG* following *LHX1* knockout. *n* = 3, one-way ANOVA. *n* = 3, unpaired *t* test. **M,** TIF of tumor cells assessed by the ELDA method following *LHX1* knockout.* n* = 8, *χ2* test. Data are shown as mean ± SD. **, *P* < 0.01; ***, *P* < 0.001; ****, *P* < 0.0001.

To investigate the role of LHX1 in HNSCC, we generated *LHX1*-knockout HNSCC cell lines and conducted RNA-seq (Fig. 2A). A total of 1,015 DEGs were identified (|log2FC| > 1, adj *P*-value < 0.05), comprising 629 upregulated and 386 downregulated DEGs (Fig. 2B). KEGG pathway analysis of downregulated DEGs demonstrated significant enrichment of CSC-associated pathways, including stem cell pluripotency regulation, Hippo signaling, and Wnt signaling (Fig. 2C). Subsequent GSEA revealed that *LHX1*-knockout suppressed biological processes linked to cell cycle, DNA replication, chromosome separation - all hallmarks of diminished self-renewal capacity in cancer cells (Supplementary Fig. S2A). The RNA-seq results strongly suggest that LHX1 plays a critical role in regulating CSC properties in HNSCC.

To validate these findings, we assessed the colony and sphere formation abilities in *LHX1*-knockout CAL27 and FaDu cells (endogenous high LHX1 expressers) and LHX1-overexpressing SCC25 and HN4 cells (endogenous low LHX1 expressers). The results showed that genetic ablation of *LHX1* significantly impaired both colony and sphere formation abilities (Fig. 2D, E, H, I), while ectopic LHX1 expression enhanced these functional properties (Supplementary Fig. S2B, C; Fig. 2F, G, J, K). Consistent with these phenotypic observations, CSC-related markers such as *ALDH1A1*, *SOX2*, *POU5F1*, and *NANOG*, were downregulated in *LHX1*-knockout cells, contrasted by upregulated expression of these markers in LHX1-overexpressing counterparts (Fig. 2L; Supplementary Fig. S2D). To further investigate CSC properties *in vivo*, we performed limiting dilution assays - recognized as the definitive methodology for quantifying CSC frequency based on their unique capacity for tumor initiation from minimal cell numbers^20^. This analysis demonstrated a substantial reduction in TIF for *LHX1*-knockout HNSCC cells, whereas LHX1-overexpressing cells exhibited enhanced tumorigenic potential (Fig. 2M; Supplementary Fig. S2E). These comprehensive findings provide compelling evidence that LHX1 plays a crucial role in maintaining CSC properties in HNSCC through augmentation of self-renewal capacity and tumor-initiating potential.

### LHX1 impedes SASP to preserve CSC properties

**Figure 3:**
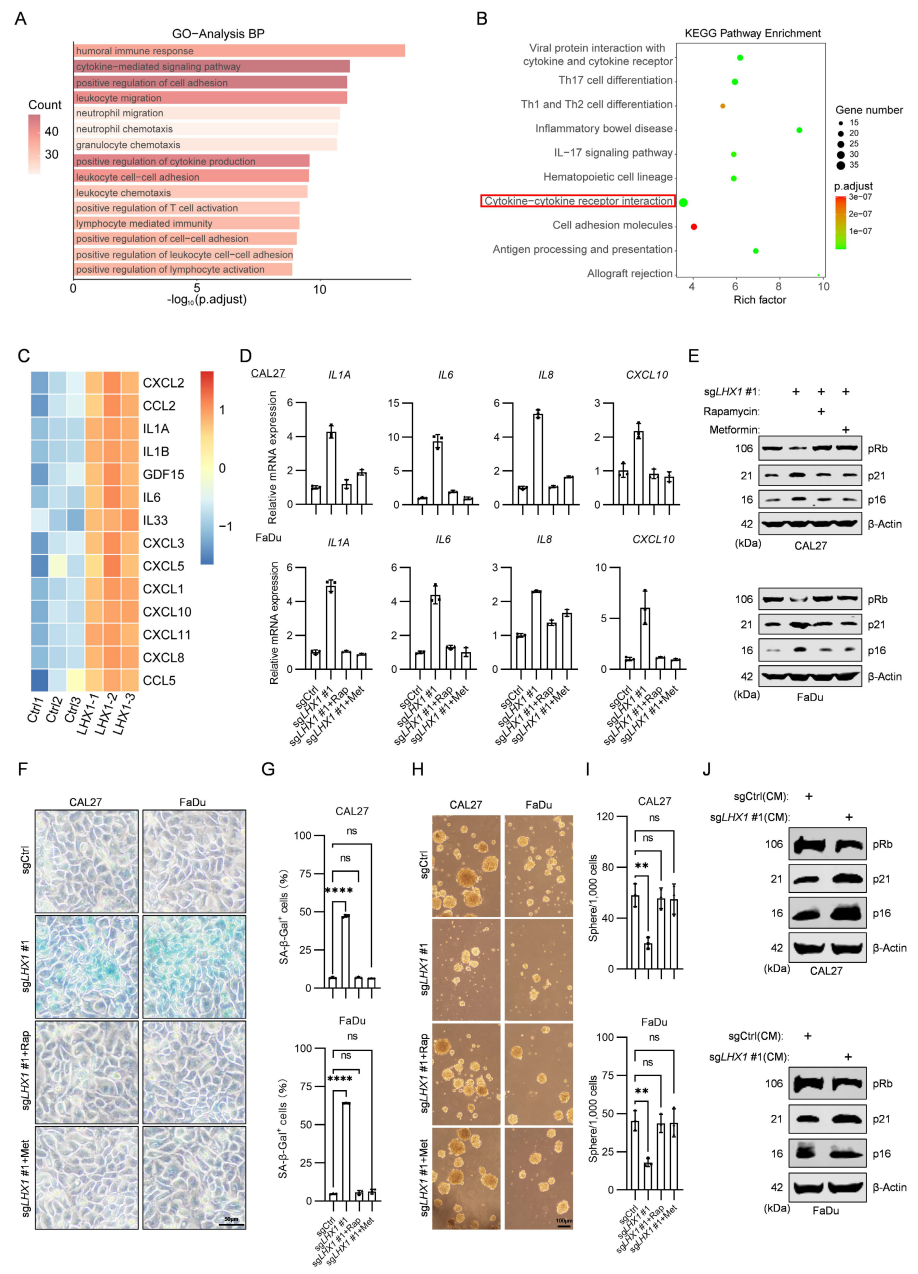
** LHX1 impedes SASP to maintain CSC properties. A,** GO analysis of 629 upregulated DEGs following *LHX1* knockout. **B,** KEGG analysis of 629 upregulated DEGs following *LHX1* knockout. **C,** Heatmap of SASP factors expression following *LHX1* knockout. **D,** RT-qPCR analysis of *IL1A, IL6, IL8,* and *CXCL10* in *LHX1*-knockout cells following treatment with SASP inhibitors. *n* = 3, one-way ANOVA. **E,** Immunoblotting of pRb, p21, and p16 in *LHX1*-knockout cells following treatment with SASP inhibitors.** F,** SA-β-gal staining in *LHX1*-knockout cells following treatment with SASP inhibitors. Scale bar, 50 μm.** G,** Quantification of the percentage of SA-β-gal positive cells.* n* = 3, one-way ANOVA. **H,** Sphere formation assay in *LHX1*-knockout cells following treatment with SASP inhibitors. Scale bar, 100 μm. **I,** Quantification of the sphere numbers. *n* = 3, one-way ANOVA. **J,** Immunoblotting of pRb, p21, and p16 in recipient cells following treatment with CM from LHX1-knockout cells. Data are shown as mean ± SD. **, *P* < 0.01; ****, *P* < 0.0001; ns, not significant.

While we have demonstrated that LHX1 contributes to the properties of CSC, the underlying mechanism remains unclear. GO and KEGG pathway analysis on 629 upregulated DEGs following *LHX1*-knockout revealed notable enrichment in cytokine-mediated signaling pathway and cytokine-cytokine receptor interaction (Fig. 3A, B). Senescent cells transmit senescence signals through the secretion of SASP factors, which activate senescence pathways and induce cell cycle arrest in receiving cells^5^. In the context of cancer, paracrine senescence can contribute to tumor suppression^39^. It remains to be determined whether LHX1 modulates SASP to regulate CSC properties. Transcriptomic profiling indicated substantial elevation of SASP components in *LHX1*-depleted cells (Fig. 3C), a finding corroborated by qPCR validation showing increased *IL1A*,* IL6*, *IL8*, and *CXCL10* expression (Fig. 3D). To directly confirm SASP factor secretion, we quantified IL-1α, IL-6 and IL-8 levels in conditioned media by ELISA. *LHX1* knockout significantly increased the secretion of all cytokines (Supplementary Fig. S3A), providing direct protein-level evidence that LHX1 suppresses SASP secretion. The upregulation of cyclin-dependent kinase (CDK) inhibitors, particularly p21 and p16, is a hallmark characteristic of senescent cells^40^. The sustained inhibition of these CDK inhibitors leads to reduced Rb phosphorylation, a critical event for cell cycle progression^41^. Next, we investigated the expression of senescence markers, and revealed an increase of p16 and p21, along with reduced pRb expression in *LHX1*-knockout cells (Fig. 3E). Moreover, SA-β-gal staining visually confirmed a significant increase in senescent cells upon *LHX1* knockout (Fig. 3F, G), providing morphological validation of the senescence induction. Conversely, LHX1-overexpressing cells displayed suppressed SASP factor expression and reversed senescence marker patterns (Supplementary Fig. S3B, C).

To establish causal relationships between SASP activation and CSC impairment, *LHX1*-knockout cells were treated with rapamycin and metformin, both known inhibitors of SASP^42^. The treatments significantly attenuated senescence induction (Fig. 3D-G; Supplementary Fig. S3A), while concurrently restoring colony/sphere formation capacities and CSC marker expression (Fig. 3H, I; Supplementary Fig. S3D-F). As SASP has the ability to induce senescence in neighboring cells through paracrine signaling, we transferred the supernatants from *LHX1*-knockout cells (with enriched SASP factors) to surrounding cancer cells. The recipient cells exhibited increased senescence markers, diminished colony and sphere formation potential, and reduced stemness markers (Fig. 3J; Supplementary Fig. S4A-E). To identify the key cytokine mediating this paracrine effect, we employed neutralizing antibodies against candidate SASP factors during *LHX1* ablation, including Bermekimab (anti-IL-1α) and Clazakizumab (anti-IL-6). We found that neutralization of IL-1α, but not IL-6, significantly attenuated senescence marker induction and restored the colony and sphere formation capacities in *LHX1*-knockout cells (Supplementary Fig. S4F-J), phenocopying the effects of rapamycin and metformin. These results provide provides direct evidence that IL-1α is a key SASP factor through which *LHX1* ablation impairs CSC properties. These complementary approaches conclusively demonstrate that LHX1 preserves CSC properties through suppressing SASP-mediated senescence signaling.

### LHX1 suppresses *STING* transcription by directly binding to its promoter

**Figure 4:**
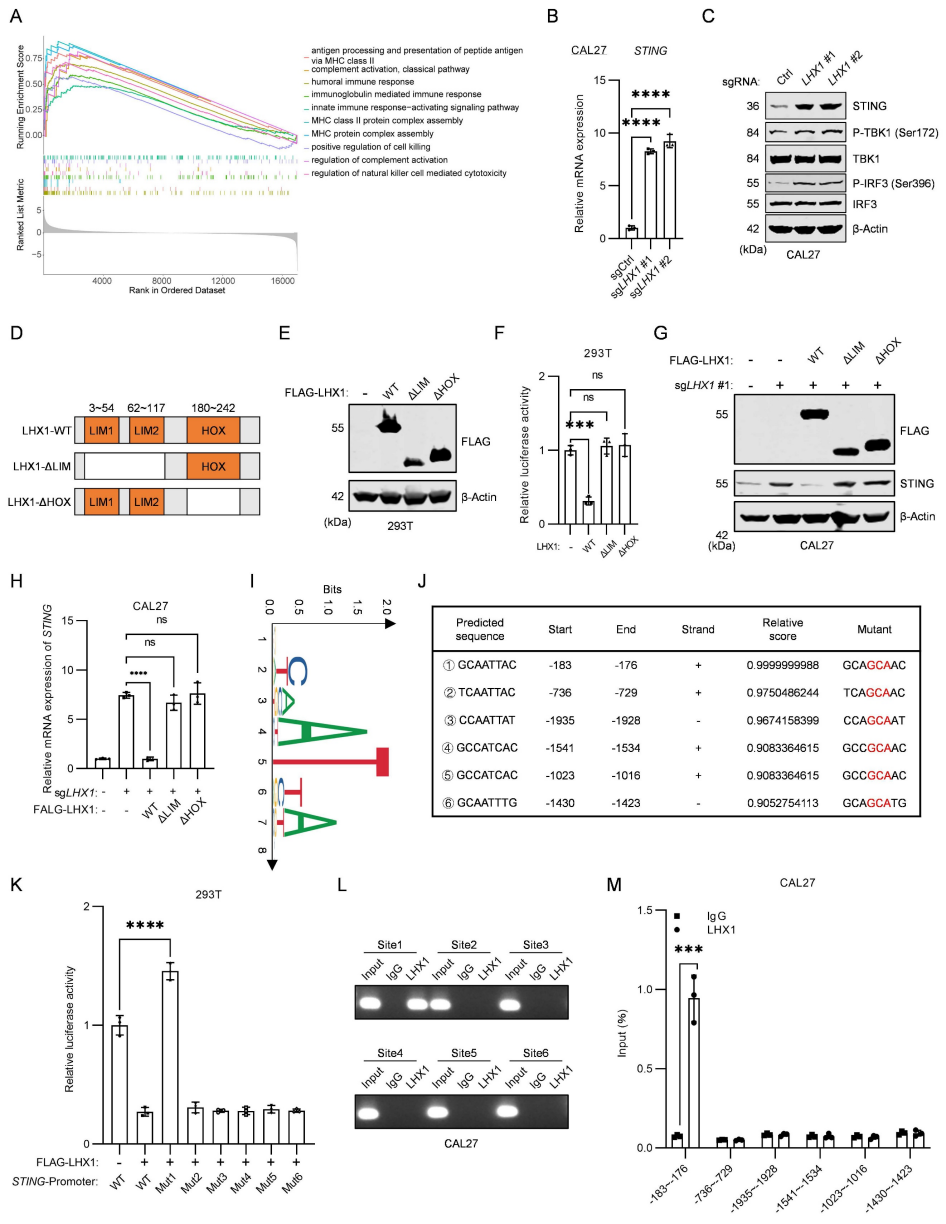
** LHX1 suppresses *STING* transcription by directly binding to its promoter. A,** GSEA was performed following *LHX1* knockout, revealing the upregulated pathways. **B,** RT-qPCR analysis of *STING* in *LHX1* knockout CAL27 cells. *n* = 3, one-way ANOVA. **C,** Immunoblotting analysis of STING, P-TBK1, TBK1, P-IRF3, and IRF3 in *LHX1* knockout CAL27 cells. **D,** Sequence diagram of the LHX1-WT, LHX1-ΔLIM, and LHX1-ΔHOX constructs. **E,** Immunoblotting of 293T cells following transfected with LHX1-WT, LHX1-ΔLIM, and LHX1-ΔHOX constructs. **F,** Dual-luciferase reporter assay evaluating the effect of LHX1-WT, LHX1-ΔLIM, and LHX1-ΔHOX on *STING* promoter activity. *n* = 3, one-way ANOVA. **G,** Immunoblotting analysis of STING following re-expression of LHX1-WT, LHX1-ΔLIM, and LHX1-ΔHOX in *LHX1* knockout CAL27 cells. **H,** RT-qPCR analysis of *STING* following re-expression of LHX1-WT, LHX1-ΔLIM, and LHX1-ΔHOX in *LHX1* knockout CAL27 cells. *n* = 3, one-way ANOVA. **I,** Identify the binding motif of LHX1 using Jaspar. **J,** Prediction of LHX1 binding sites on the *STING* promoter. **K,** Dual-luciferase reporter assay was used to evaluate the effects of LHX1 on different *STING* promoter mutants. *n* = 3, one-way ANOVA. **L,** ChIP-PCR analysis of LHX1 binding to different regions of the *STING* promoter in CAL27 cells. **M,** ChIP-qPCR analysis of LHX1 binding to different regions of the *STING* promoter in CAL27 cells. *n* = 3, unpaired *t* test. Data are shown as mean ± SD. **, *P* < 0.01; ***, *P* < 0.001; ****, *P* < 0.0001; ns, not significant.

cGAS is responsible for recognizing free dsDNA in the cytoplasm and catalyzing the synthesis of the second messenger, cGAMP, which binds to the cytoplasmic ligand-binding domain of STING, thereby activating STING through a conformational change^43^. The cGAS-STING pathway not only mediates antiviral immunity but also participates in SASP regulation^44^,^45^. GSEA of *LHX1*-depleted cells revealed marked activation of innate immunity pathways, including antigen processing and presentation of peptide antigen via MHC class Ⅱ, humoral immune response, and MHC protein complex assembly (Fig. 4A). These findings aligned with GO and KEGG analyses of upregulated DEGs (Fig. 3A, B), suggesting LHX1 suppresses SASP while modulating innate immunity. Given the central role of the cGAS-STING pathway in both innate immunity and SASP, we hypothesized LHX1 might govern this pathway. Transcriptome analysis demonstrated selective upregulation of *STING* expression upon *LHX1* knockout without affecting *cGAS* levels (Supplementary Fig. S5A). To validate this, we examined the expression of cGAS and STING, confirming that* LHX1* knockout enhanced STING and activated its downstream pathways, including an increase in the expression levels of P-TBK1 and P-IRF3, while LHX1 overexpression inhibited STING expression (Fig. 4B, C; Supplementary Fig. S5B-E). Furthermore, neither *LHX1* knockout nor overexpression had a significant effect on cGAS level (Supplementary Fig. S5F, G). To determine whether LHX1 additionally modulates upstream pathway activation, we measured intracellular cGAMP levels. While basal cGAMP levels in HNSCC cells were elevated compared to normal epithelial cells, neither *LHX1* knockout nor overexpression significantly altered cGAMP levels (Supplementary Fig. S5H-J), indicating that LHX1 does not affect cytosolic dsDNA abundance. This supports the conclusion that LHX1 regulates the STING pathway primarily at the level of receptor transcription.

LHX1 is a transcription factor with two LIM domains and a HOX domain. The LIM domains mediate protein interactions, while the HOX domain binds directly to DNA regions of target genes^46^. To investigate whether LHX1 transcriptionally regulates *STING*, we constructed wild-type LHX1 (LHX1-WT), a mutant lacking the LIM domains (LHX1-ΔLIM), and a mutant lacking the HOX domain (LHX1-ΔHOX), and co-transfected these constructs into HEK293T cells with a pGL4 luciferase plasmid containing the *STING* promoter and a phRL-TK plasmid (Fig. 4D, E). LHX1-WT significantly inhibited the transcriptional activity of* STING*, while both LHX1-ΔLIM and LHX1-ΔHOX lost this inhibitory effect (Fig. 4F). To further assess the importance of LHX1's transcriptional activity in repressing *STING*, we reintroduced LHX1-WT, LHX1-ΔLIM, and LHX1-ΔHOX into *LHX1*-depleted cells. The results showed that both the LHX1-ΔHOX mutant, lacking the DNA-binding region, and the LHX1-ΔLIM mutant, lacking the protein interaction domain, failed to repress STING expression (Fig. 4G, H; Supplementary Fig. S5K, L). These findings strongly suggest that LHX1 requires interaction with other auxiliary proteins to form a transcriptional complex necessary to repress *STING* transcription. Subsequently, JASPAR was employed to ascertain the motif of LHX1 and to predict the potential sites at which LHX1 binds to the promoter region of *STING* (Fig. 4I, J). We then constructed various mutants of the *STING* promoter, and dual luciferase assays showed that LHX1 binds to the region from -183 to -176 of the *STING* promoter. Mutating this DNA sequence abolished LHX1's inhibition of *STING* transcription (Fig. 4K). ChIP-PCR and ChIP-qPCR experiments further confirmed that LHX1 bound to the -183 to -176 region of the *STING* promoter (Fig. 4L, M; Supplementary Fig. S5M, N). These data demonstrate that LHX1 directly binds to the *STING* promoter and represses its transcription. Having established that LHX1 directly binds to the *STING* promoter, we sought to delineate the specific epigenetic mechanism by which it mediates transcriptional repression. Analysis of TCGA data revealed that *STING* promoter DNA methylation was lower in HNSCC tumors compared to normal tissues, inconsistent with being the primary repressive mechanism (Supplementary Fig. S6A, B). We therefore investigated repressive histone modifications. ChIP-qPCR analysis demonstrated that *LHX1* knockout specifically led to a significant decrease in the enrichment of the repressive histone mark H3K9me3, but not H3K27me3, at the *STING* promoter (Supplementary Fig. S6C). These findings indicate that the LHX1-LDB1 complex transcriptionally represses *STING* by specifically facilitating the deposition of H3K9me3 onto its promoter. To further substantiate that LHX1 functions through STING, we treated cells with the STING agonist cGAMP. cGAMP treatment phenocopied the effects of *LHX1* knockout, leading to enhanced senescence marker expression and impaired clonogenic capacity (Supplementary Fig. S6D-F). Combined with our finding that *LHX1* knockout does not alter cGAMP levels, this provides strong pharmacological evidence that LHX1 regulates the STING-SASP axis by controlling STING receptor abundance rather than modulating upstream agonist availability.

### STING is involved in the LHX1-mediated regulation of SASP

**Figure 5:**
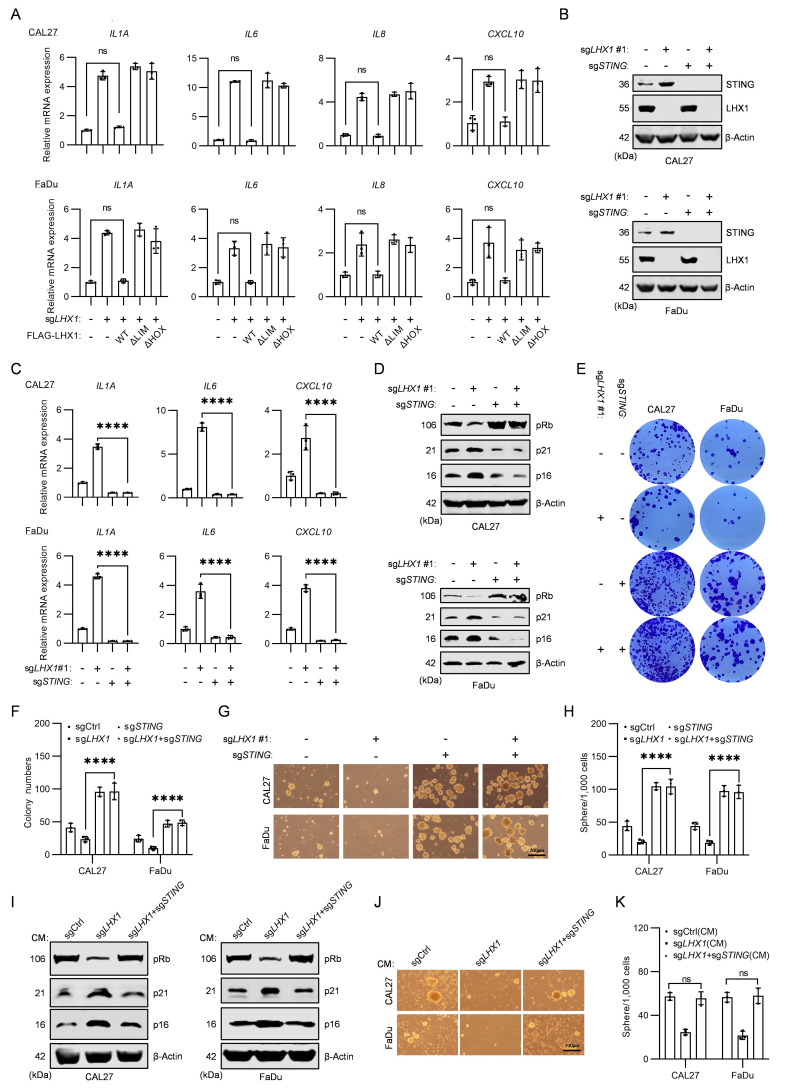
** STING is involved in the LHX1-mediated regulation of SASP. A,** RT-qPCR analysis of *IL1A, IL6, IL8* and *CXCL10* following re-expression of LHX1-WT, LHX1-ΔLIM and LHX1-ΔHOX in *LHX1*-knockout cells. *n* = 3, one-way ANOVA. **B,** Immunoblotting analysis of LHX1 and STING in* LHX1* and *STING* double knockout cells. **C,** RT-qPCR analysis of *IL1A, IL6,* and *CXCL10* in *LHX1* and *STING* double knockout cells. *n* = 3, one-way ANOVA. **D,** Immunoblotting analysis of pRb, p21, and p16 in *LHX1* and *STING* double knockout cells. **E,** Colony formation assay in *LHX1* and *STING* double knockout cells. **F,** Quantification of the colony numbers. *n* = 3, one-way ANOVA. **G,** Sphere formation assay in *LHX1* and *STING* double knockout cells. Scale bar, 200 μm. **H,** Quantification of the sphere numbers in *LHX1* and *STING* double knockout cells. *n* = 3, one-way ANOVA. **I,** Immunoblotting analysis of pRb, p21, and p16 in recipient cells following treatment with CM from *LHX1*-knockout cells or *LHX1* and *STING* double knockout cells.** J,** Sphere formation assay in recipient cells following treatment with CM from *LHX1*-knockout cells or *LHX1* and *STING* double knockout cells. Scale bar, 200 μm. **K,** Quantification of the sphere numbers in recipient cells.* n* = 3, one-way ANOVA. Data are shown as mean ± SD. ****, *P* < 0.0001; ns, not significant.

To determine whether STING is a downstream target of LHX1 in regulating SASP and CSC properties, we examined the impacts of LHX1-WT, LHX1-ΔLIM, and LHX1-ΔHOX mutants on SASP-associated factors. Only LHX1-WT effectively suppressed SASP (Fig. 5A), consistent with previous results showing that only LHX1-WT repressed STING expression. We next performed concurrent *STING* knockout in *LHX1*-depleted cells and re-evaluated SASP-related factor expression (Fig. 5B). The results showed that the enhanced SASP phenotype induced by *LHX1* knockout was fully reversed by *STING* depletion, indicating that LHX1-mediated regulation of SASP was dependent on STING (Fig. 5C). Additionally, the upregulation of p16 and p21, as well as the downregulation of pRb following *LHX1* knockout, were all reversed upon *STING* depletion (Fig. 5D). Moreover, *STING* knockout also reversed the impaired colony and sphere formation capacities of cancer cells resulting from *LHX1* knockout (Fig. 5E-H). We previously showed that the increased SASP factors in the CM of *LHX1*-depleted cancer cells induced cellular senescence and reduced CSC properties in neighboring cancer cells. To investigate whether *STING* knockout also affected these secreted factors, we analyzed the supernatants from *LHX1*- and *STING*-depleted cancer cells. The results demonstrated that *STING* knockout abolished the effects of *LHX1* knockout supernatants on the senescent state and CSC properties of surrounding cells (Fig. 5I-K, Supplementary Fig. S7A, B). Notably, even in *STING*-knockout cells, an increase in senescence markers (p16 and p21) and a decrease in CSC properties were still evident upon exposure to CM enriched with SASP factors (Supplementary Fig. S7C-G). This suggests that while SASP secretion is significantly influenced by STING, its biological activity does not rely on STING. Collectively, these results indicate that STING is a downstream target of LHX1 and is essential for modulating SASP.

### LDB1 is indispensable for LHX1-targeted regulation of STING

**Figure 6:**
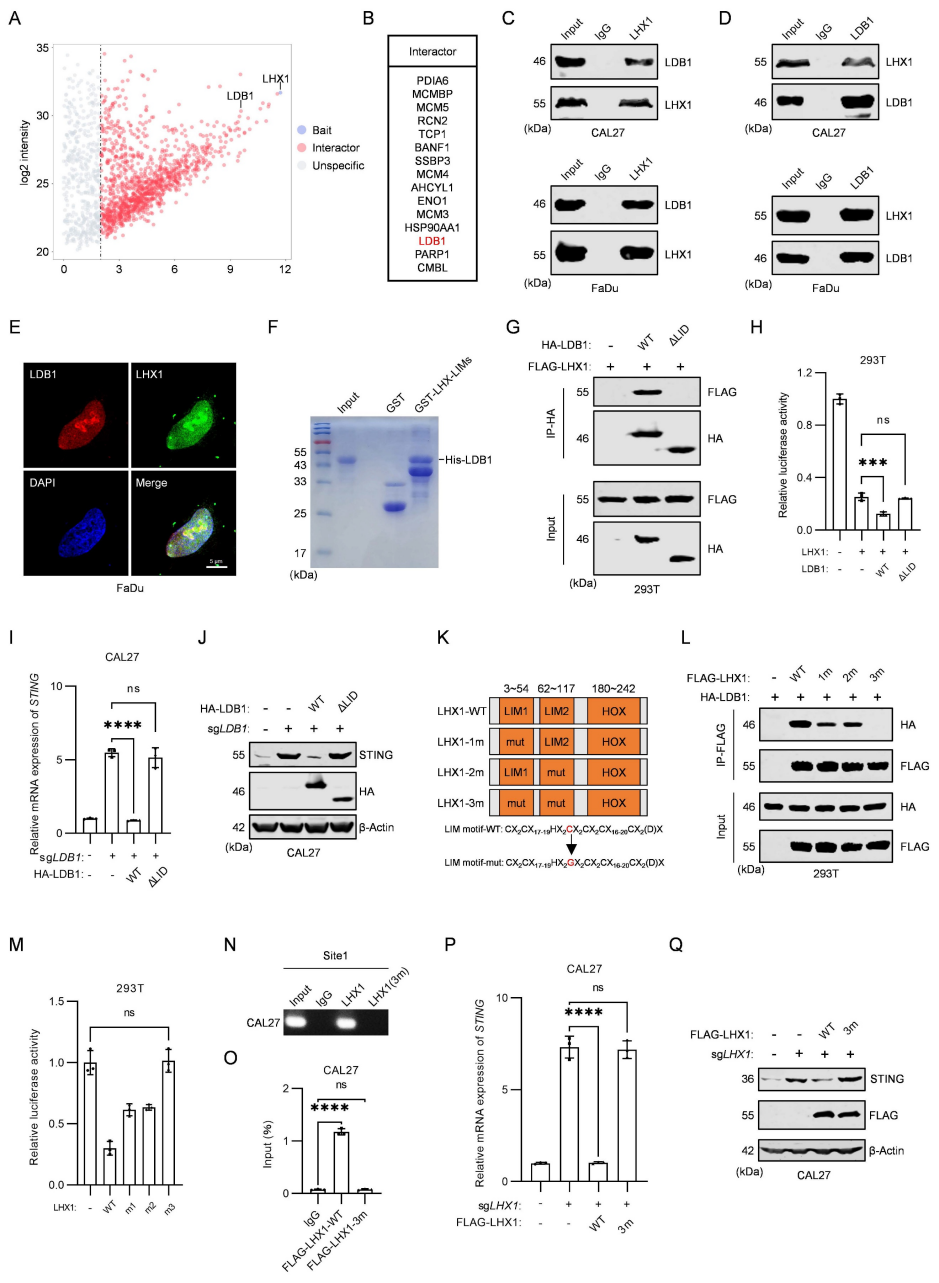
** LDB1 is indispensable for LHX1-targeted regulation of STING. A,** IP-MS identified proteins interacting with LHX1 in FaDu cells. **B,** Top 15 proteins interacting with LHX1. **C,** Co-IP results of LHX1 and LDB1 in HNSCC cells, with LHX1 serving as the bait protein. **D,** Co-IP results of LDB1 and LHX1 in HNSCC cells, with LDB1 serving as the bait protein. **E,** IF staining of LDB1 and LHX1 in FaDu cells. Scale bar, 5 μm. **F,** GST-pull down assay examining the interaction between His-LDB1 and GST-LHX1-LIMs. **G,** Co-IP results of FLAG-LHX1 and HA-LDB1 mutants in 293T cells.** H,** Dual-luciferase reporter assay evaluating the roles of LDB1-WT and LDB1-ΔLID mutant on *STING* promoter activity. *n* = 3, one-way ANOVA. **I,** RT-qPCR analysis of* STING* following re-expression of LDB1-WT and LDB1-ΔLID mutant in *LDB1*-knockout CAL27 cells. *n* = 3, one-way ANOVA. **J,** Immunoblotting analysis of STING following re-expression of LDB1-WT and LDB1-ΔLID mutant in *LDB1*-knockout CAL27 cells. **K,** Sequence diagram of the LHX1-WT, LHX1-1m, LHX1-2m and LHX1-3m mutants. **L,** Co-IP results of HA-LDB1 and FLAG-LHX1 mutants in 293T cells, with FLAG-LHX1 serving as the bait protein. **M,** Dual-luciferase reporter assay evaluating the effect of LHX1-WT, LHX1-1m, LHX1-2m, and LHX1-3m mutants on *STING* promoter activity. *n* = 3, one-way ANOVA.** N,** ChIP-PCR analysis of the binding of LHX1-WT and LHX1-3m mutant to the site1 region of *STING* promoter in CAL27 cells. **O,** ChIP-qPCR analysis of the binding of LHX1-WT and LHX1-3m mutant to the site1 region of *STING* promoter in CAL27 cells. *n* = 3, one-way ANOVA. **P,** RT-qPCR analysis of* STING* following re-expression of LHX1-WT or LHX1-3m mutant in *LHX1* knockout CAL27 cells. *n* = 3, one-way ANOVA. **Q,** Immunoblotting analysis of STING following re-expression of LHX1-WT or LHX1-3m mutant in *LHX1* knockout CAL27 cells. Data are shown as mean ± SD. ***, *P* < 0.001; ****, *P* < 0.0001; ns, not significant.

It has been shown that LHX1 lacking the LIM domain is incapable of targeting and regulating *STING*, suggesting that LHX1-interacting proteins are critical for its repression of *STING* transcription. To identify the key auxiliary interactors involved in this process, we performed IP-MS experiments to search for LHX1-binding proteins (Fig. 6A). Among the top 15 enriched proteins, LIM domain-binding protein 1 (LDB1) was identified (Fig. 6B). LDB1 is a transcriptional co-regulator that interacts with the LIM domain and is involved in regulating various biological processes^47^-^49^. However, its role in *STING* transcriptional regulation has not been previously explored. In cells overexpressing LDB1, STING expression decreased, whereas in *LDB1*-knockout cells, STING expression increased (Supplementary Fig. S8A-D). These effects mirrored those observed with LHX1 overexpression or knockout. Next, we performed Co-IP experiments in both HEK293T cells and HNSCC cells, confirming the interaction between LHX1 and LDB1 (Fig. 6C, D; Supplementary Fig. S8E, F). IF results showed that both LDB1 and LHX1 are predominantly localized in the nucleus, with significant co-localization observed within the nuclear compartment (Fig. 6E). Furthermore, we purified GST-LHX1-LIMs and His-LDB1 proteins and conducted *in vitro* GST-pulldown assays, which provided additional evidence for the interaction between LHX1 and LDB1 (Fig. 6F).

The LID domain of LDB1 is indispensable for its binding to the LIM domain^28^. We observed that the LDB1-ΔLID mutant failed to interact with LHX1 (Supplementary Fig. S8G; Fig. 6G). Dual luciferase assays revealed that only the LDB1-WT construct enhanced LHX1-mediated transcriptional repression of *STING*, with no effect in the LDB1-ΔLID mutant (Fig. 6H). To validate these results, we re-expressed LDB1-WT and LDB1-ΔLID in *LDB1*-knockout cells. Only LDB1-WT was able to restore repression of STING expression, supporting the notion that LDB1 must bind to LHX1 to facilitate its function (Fig. 6I, J; Supplementary Fig. S8H, I). Previous studies have shown that a mutation in the third cysteine of the LIM domain in LHX1 prevents binding to LDB1^50^. We generated LHX1 mutants (LHX1-1m, LHX1-2m, and LHX1-3m) and found that LHX1-3m completely lost its interaction with LDB1 (Fig. 6K, L). Dual luciferase assays revealed that LHX1-3m could not repress *STING* transcription (Fig. 6M). ChIP-PCR and ChIP-qPCR experiments demonstrated that LHX1-3m was unable to bind the *STING* promoter (Fig. 6N, O; Supplementary Fig. S8J, K). Re-expression of LHX1-WT and LHX1-3m in *LHX1*-knockout cells showed that the LHX1-3m mutant, unable to interact with LDB1, failed to inhibit STING expression (Fig. 6P, Q; Supplementary Fig. S8L, M). These results confirm that the interaction between LHX1 and LDB1 is essential for LHX1-mediated regulation of STING.

### Blocking the interaction between LDB1 and LHX1 inhibits HNSCC progression

**Figure 7:**
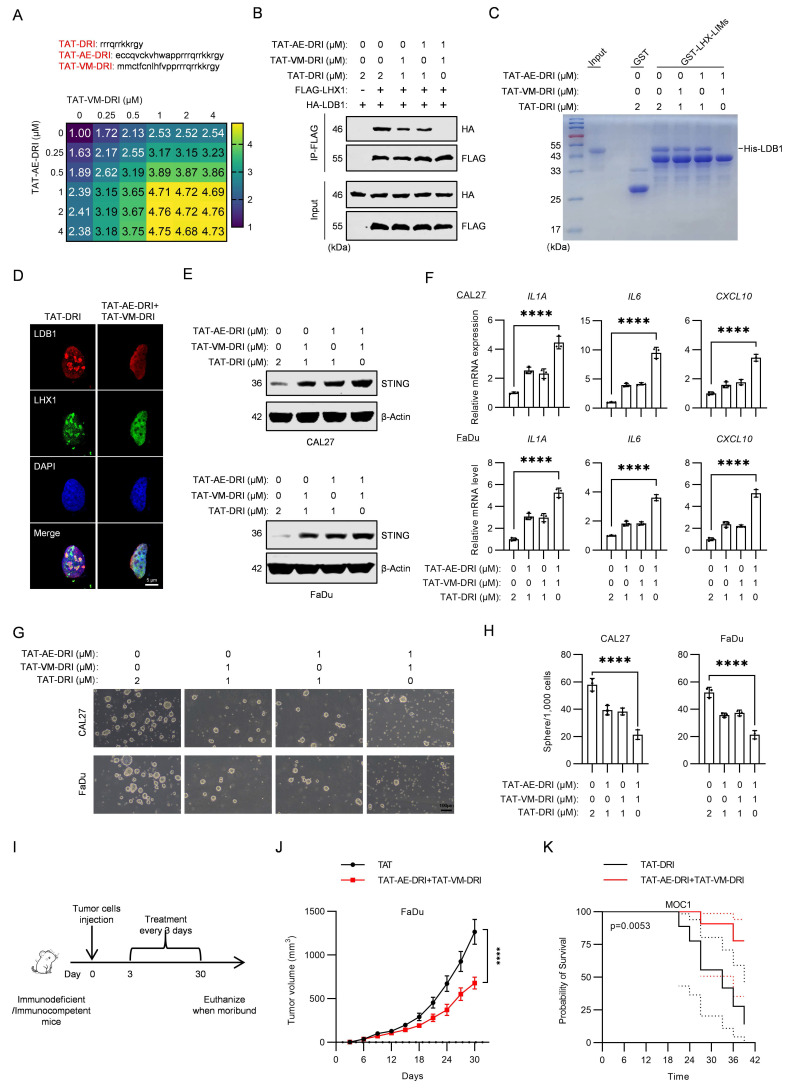
** Blocking the interaction between LDB1 and LHX1 inhibits HNSCC progression. A,** RT-qPCR analysis of *STING* expression in FaDu cells when treated with different concentrations of TAT-AE-DRI and TAT-VM-DRI peptides. **B,** Co-IP results of HA-LDB1 and FLAG-LHX1 in 293T cells when treated with TAT-AE-DRI and TAT-VM-DRI peptides. **C,** GST pull-down assay assessing the interaction between His-LDB1 and GST-LHX1-LIMs in the presence of TAT-AE-DRI and TAT-VM-DRI peptides. **D,** IF staining of LDB1 and LHX1 in FaDu cells with or without TAT-AE-DRI and TAT-VM-DRI peptides. Scale bar, 5 μm. **E,** Immunoblotting analysis of STING in HNSCC cells when treated with TAT-AE-DRI and TAT-VM-DRI peptides. **F,** RT-qPCR analysis of* IL1A*,* IL6,* and *CXCL10* following treatment with TAT-AE-DRI and TAT-VM-DRI peptides. *n* = 3, one-way ANOVA. **G,** Sphere formation assay following treatment with TAT-AE-DRI and TAT-VM-DRI peptides. Scale bar, 100 μm. **H,** Quantification of the sphere numbers. *n* = 3, one-way ANOVA. **I,** Tumor cells were inoculated, followed by intraperitoneal 1 μmol/kg TAT-AE-DRI and 1 μmol/kg TAT-VM-DRI peptides injection every 3 days, with tumor size and mouse survival being recorded. **J,** Effect of TAT-AE-DRI and TAT-VM-DRI peptides on the growth of FaDu-derived subcutaneous xenografts.* n* = 8, unpaired *t* test. **K,** Impact of TAT-AE-DRI and TAT-VM-DRI peptides on the survival of mice in the MOC1 orthotopic tongue tumor model. *n* = 8, Kaplan-Meier curve. Data are shown as mean ± SD. ****, *P* < 0.0001.

**Figure 8:**
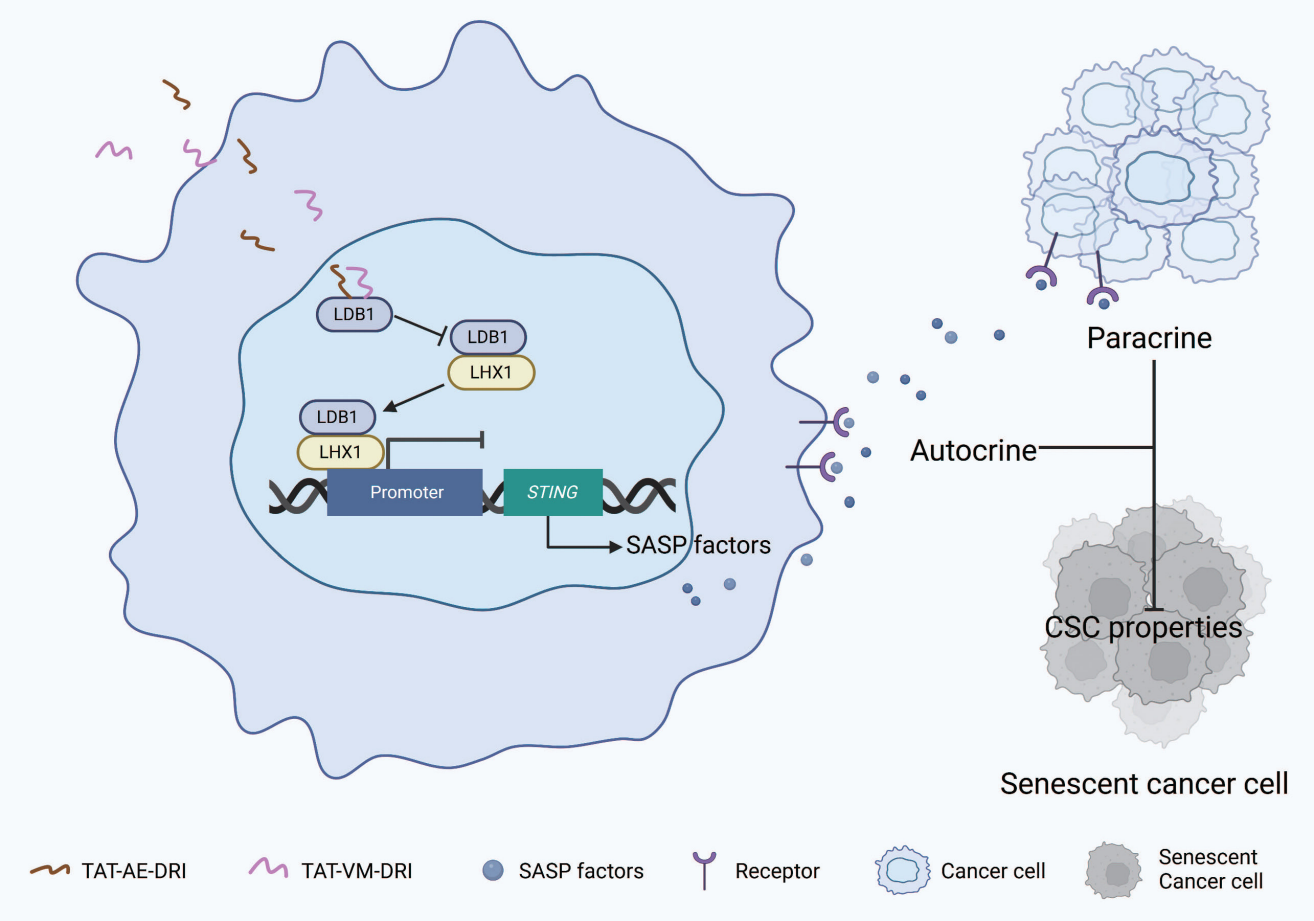
** Schematic model of TAT-AE-DRI and TAT-VM-DRI peptides disrupting the LDB1-LHX1 interaction to suppress HNSCC progression.** The TAT-AE-DRI and TAT-VM-DRI peptides bind to LDB1, thereby blocking the formation of the LHX1-LDB1 transcriptional complex. This alleviates the repression of STING, restores SASP activity, and induces cancer cell senescence through paracrine and autocrine mechanisms. Consequently, CSC properties are impaired, leading to suppression of HNSCC progression.

Given that mutation of the third cysteine in the LIM domain of LHX1 prevents its binding to LDB1, we hypothesized that the amino acid sequence surrounding this cysteine might be critical for the interaction between LHX1 and LDB1. To test this, we designed two peptides, AE and VM, to mimic the relevant region of the sequence (Supplementary Fig. S9A). These peptides were expected to competitively bind to LDB1, thereby disrupting the LHX1-LDB1 interaction and inhibiting LHX1's repression of STING. Both AE and VM peptides were conjugated to the TAT peptide, a well-known cell-penetrating peptide, to facilitate cellular uptake^51^. To directly confirm the nuclear localization of these therapeutic peptides, we performed fluorescence microscopy using GFP-conjugated peptides. The results confirmed that both TAT-AE-GFP and TAT-VM-GFP efficiently enter cells and localize to the nucleus, unlike the control GFP protein (Supplementary Fig. S9B), providing direct visual evidence of their ability to access the nuclear LHX1-LDB1 complex. We evaluated the effects of these peptides on LHX1 function by measuring *STING* transcription levels. The results showed that both TAT-AE and TAT-VM effectively promoted *STING* transcription in a dose-dependent manner. Peak transcriptional activation of *STING* occurred at 2 μM for both peptides, indicating complete disruption of the LDB1-LHX1 interaction at these concentrations (Supplementary Fig. S9D). In contrast, TAT alone, used as a negative control, had no effect on *STING* transcription at similar concentrations (Supplementary Fig. S9C). Furthermore, concurrent treatment with TAT-AE and TAT-VM completely blocked the interaction between LHX1 and LDB1 and promoted STING expression (Supplementary Fig. S9E-G). To verify the specificity of these peptides for the LHX1-LDB1 interaction, we performed co-immunoprecipitation assays. The results demonstrate that our therapeutic peptides selectively disrupt LHX1-LDB1 binding without affecting the interaction between LDB1 and other LIM-domain proteins such as LMO1 or LMO2 (Supplementary Fig. S9H), confirming their high specificity. To optimize the peptides further, their sequences were reversed, and the amino acids were substituted with D-isomers to enhance their stability and potency both *in vitro* and *in vivo*^52^. The resulting peptides, designated TAT-AE-DRI and TAT-VM-DRI, exhibited stronger *STING* transcription-promoting activity and effectively disrupted the LHX1-LDB1 interaction at lower concentrations (1 μM for both TAT-AE-DRI and TAT-VM-DRI) (Fig. 7A-D). Additionally, the combination of these peptides significantly reduced STING expression and inhibited the SASP (Fig. 7E, F). We next assessed the impact of these peptides on CSC properties. The combination of TAT-AE-DRI and TAT-VM-DRI maximized the inhibition of colony formation and sphere formation (Fig. 7G, H; Supplementary Fig. S9I, J). Finally, by establishing subcutaneous xenograft tumor models in immunodeficient mice and orthotopic tongue tumor models in immunocompetent mice, we demonstrated that the combination of these peptides markedly inhibited tumor growth and prolonged mice survival (Fig. 7I-K; Supplementary Fig. S10A, B). To validate the *in vivo* mechanism of action, we performed IHC staining on harvested tumor tissues. Peptide treatment led to reduced Ki67 (proliferation), upregulation of STING, p-TBK1, and p-IRF3, confirming *in vivo* target engagement and pathway activation (Supplementary Fig. S10C, D). However, no significant change in Cleaved Caspase-3 was observed (Supplementary Fig. S10C, D), suggesting that the primary mechanism of tumor suppression is growth arrest via senescence rather than apoptosis induction. Collectively, our results suggest that the peptides disrupting the LDB1-LHX1 interaction can effectively inhibit the oncogenic function of LHX1 and suppress HNSCC progression (Fig. 8).

## Discussion

Identifying regulatory factors that contribute to the CSC properties holds significant potential for developing novel strategies to target CSC and block cancer progression. In this study, we demonstrate that LHX1 is upregulated in HNSCC and correlates with poor clinical outcomes. Mechanistic investigations reveal that LHX1 represses the SASP by binding to the promoter region of *STING*, thus maintaining CSC properties. Further studies show that the interaction between LDB1 and LHX1 plays a crucial role in LHX1-mediated repression of *STING* transcription. Inhibition of this interaction using specific peptides effectively suppresses the CSC characteristics and inhibits HNSCC progression. These findings suggest that LHX1 regulates CSC traits by modulating STING-driven SASP, providing a potential therapeutic strategy for HNSCC.

LHX1 is a crucial transcription factor involved in organogenesis during embryonic development^29^. Cervino *et al.* demonstrated that Ldb1-Lhx1-Ssbp transcriptional complex plays a role in renal organogenesis during embryonic development^53^. Sibbritt *et al.* found that loss of LHX1 function leads to anterior truncation in the embryo, resulting from disrupted morphogenetic movements of tissue precursors and dysregulation of WNT signaling^54^. While the role of LHX1 in organogenesis is well-documented, its involvement in tumor biology, especially in CSC regulation, remains poorly understood. Habib *et al.* identified LHX1 as a potential biomarker for HNSCC, noting that its expression correlated with reduced survival in HNSCC patients, though its functional role in HNSCC was not fully explored^55^. Our study provides compelling evidence that LHX1 is elevated in HNSCC and correlates with advanced clinicopathological features. Elevated LHX1 expression is associated with poorer prognosis in HNSCC patients. An important and unresolved question is what drives the upregulation of LHX1 in HNSCC. While beyond the scope of this study, existing evidence points to several potential mechanisms operating at genetic, epigenetic, and transcriptional levels^56^-^58^. The multifactorial nature of LHX1 upregulation warrants further investigation in HNSCC, particularly through analysis of larger patient cohorts for genetic alterations and detailed mechanistic studies on its transcriptional and epigenetic control. Furthermore, modulating LHX1 expression—either by knockout or overexpression—significantly alters CSC properties in cancer cells, supporting its potential as a diagnostic and prognostic marker in HNSCC. Interestingly, LHX1 expression also marks the most undifferentiated spermatogonial stem cells in the developing mouse testis and is regulated by niche factors that govern spermatogonial stem cell self-renewal and proliferation^59^,^60^. This suggests that LHX1 may play a broader role in the maintenance of stem cells across various tissues, in addition to its role in CSC.

Cellular senescence, typically characterized by cell cycle arrest, contrasts with the unlimited self-renewal capacity of CSC^61^,^62^. Based on this, we hypothesized that LHX1 may preserve CSC properties by inhibiting senescence. RNA-seq analysis revealed that knockout of *LHX1* significantly activated the SASP, a hypersecretory state of senescent cells that promotes peripheral senescence through both autocrine and paracrine signaling.

Our study also shows that transferring supernatants from *LHX1*-knockout cells, enriched with SASP factors, to surrounding cancer cells induces senescence in recipient cells and compromises their CSC characteristics. These findings validate our hypothesis and provide further insights into the role of LHX1 in modulating HNSCC progression. The STING pathway, a key cytoplasmic DNA sensing mechanism, activates innate immunity by recognizing cytoplasmic DNA^63^. Recent studies have shown that following nuclear membrane disruption, a significant amount of chromatin fragments leaks into the cytoplasm. These cytoplasmic chromatin fragments (CCFs) play a critical role in SASP development by activating the STING pathway^44^. In this study, we identify LHX1 as a novel transcription factor that directly binds to the *STING* promoter region, repressing its transcription. This repression inhibits STING-mediated activation of the SASP, leading to impaired CSC properties in both cancer cells and their surrounding counterparts. Previous studies have shown that cellular senescence can be a double-edged sword in tumors. On one hand, cellular senescence can be used as a strategy to inhibit tumor growth by inducing tumor cell senescence or selectively eliminating senescent cells. On the other hand, persistent senescent cells in the tumor microenvironment promote tumor progression, invasiveness, and treatment resistance^64^. In this context, our study demonstrates that in HNSCC, SASP factors play a role in inhibiting CSC properties and suppressing tumor growth. This underscores the complex interplay between senescence and cancer progression.

Transcription factors bind specifically to DNA sequences in target genes and require interaction with cofactors to form transcription complexes that regulate gene expression under specific conditions^65^. In their active state, LIM-HD proteins interact with LDB1 through its C-terminal LID domain, enabling tetramer formation. This interaction exposes the homeodomain of LIM-HD, enabling binding to DNA within promoter or enhancer regions to regulate gene transcription^66^. For instance, Monahan *et al.* demonstrated that Lhx2/Ldb1-mediated trans interactions are crucial for regulating olfactory receptor choice in mice^67^. In our study, we identified LDB1 as an interacting protein of LHX1. We found that the LID domain of LDB1 and the LIM domain of LHX1 directly interact, which is essential for LHX1's regulation of target gene transcription. Deletion of the LID domain in LDB1 or a mutation at the third cysteine residue of the LIM domain in LHX1 abolishes this interaction, leading to the loss of LHX1's ability to repress *STING* transcription. Furthermore, targeting the LIM domain of LHX1 with targeted peptides effectively disrupts LHX1-mediated repression of STING, inhibiting the promotion of CSC properties and demonstrating promising therapeutic effects in HNSCC.

Despite the valuable insights provided by this study, several limitations should be acknowledged. Firstly, we exclusively used cell lines and did not incorporate advanced organoid models, which better mimic the *in vivo* tumor microenvironment and offer a more accurate representation of tumor biology. Additionally, while our data from immunodeficient models establish a cell-intrinsic mechanism for tumor suppression, the potential contribution of STING-dependent, immune-mediated killing mechanisms such as T-cell infiltration and activation in an immunocompetent setting remains largely unexplored, which represents a crucial and exciting avenue for future investigation. Furthermore, the use of conditional knockout mice for *LHX1* in combination with spontaneous tumorigenesis would offer a more comprehensive validation of the tumor-promoting function of LHX1. Nonetheless, our study demonstrates that LHX1 regulates STING expression to inhibit the SASP, thereby enhancing the CSC properties and promoting HNSCC progression. Importantly, the LHX1-targeting peptides, designed based on the amino acid sequence of the LIM domain, effectively disrupt LHX1 function both *in vitro* and *in vivo*, resulting in the inhibition of HNSCC progression. These findings contribute to a deeper understanding of the molecular mechanisms underlying CSC and offer valuable insights for the rational design of targeted anti-HNSCC therapies.

## Supplementary Material

Supplementary figures and tables.

## Abbreviations

SASP: Senescence-associated secretory phenotype
LHX1: LIM homeobox 1
HNSCC: Head and neck squamous cell carcinoma
IHC: Immunohistochemical
IP-MS: Immunoprecipitation followed by mass spectrometry
Co-IP: Co-immunoprecipitation
STING: Stimulator of interferon genes
CSC: Cancer stem cells
EMT: Epithelial-mesenchymal transition
TCGA: The Cancer Genome Atlas
GEO: Gene Expression Omnibus
RNA-seq: RNA sequencing
DEGs: Differentially expressed genes
GO: Gene Ontology
KEGG: Kyoto Encyclopedia of Genes and Genomes
GSEA: Gene Set Enrichment Analysis
IF: Immunofluorescence
ELDA: Extreme limiting dilution assay
TIF: Tumor-initiating frequency
OS: Overall survival
PFI: Progression-free interval
DFI: Disease-free interval
DSS: Disease-specific survival
CDK: Cyclin-dependent kinase
CCFs: Cytoplasmic chromatin fragments
