# Supplementary material for: Targeting the LHX1-LDB1 Complex Restores STING-dependent Senescence Surveillance and Inhibits Head and Neck Cancer Progression
Source: Int J Biol Sci. 2026 Jan 1;22(3):1186–206. doi: 10.7150/ijbs.123790 (PMC12836506; doi:10.7150/ijbs.123790)
Supplement: Supplementary file 1 — Supplementary figures and tables. [file ijbsv22p1186s1.pdf]

Supplementary Figure 1

A, Correlation between LHX1 expression and DFI in HNSCC patients. **B,** Correlation between LHX1 expression and DSS in HNSCC patients. **C,** Correlation between LHX1 expression and tumor size in HNSCC patients. **D,** Correlation between LHX1 expression and lymph node metastasis in HNSCC. **E,** Correlation between LHX1 expression and distant metastasis in HNSCC patients. **F,** Correlation between LHX1 expression and clinical stage in HNSCC. **G,** Correlation of LHX1 expression with pathological grade in HNSCC. **H,** Correlation between LHX1 expression and age in HNSCC patients. **I,** Correlation between LHX1 expression and gender in HNSCC patients. *, $P < 0.05$; **, $P < 0.01$; ***, $P < 0.001$; ****, $P < 0.0001$; ns, not significant.

Supplementary Figure 2

A, GSEA was performed following *LHX1* knockout, revealing the downregulated pathways. **B,** Immunoblotting of LHX1 following the LHX1 overexpression. **C,** RT-qPCR analysis of *LHX1* following LHX1 overexpression. $n = 3$, unpaired t test. **D,** RT-qPCR analysis of *ALDH1A1, SOX2, POU5F1,* and *NANOG* following LHX1 overexpression. **E,** TIF of tumor cells assessed by the ELDA method following LHX1 overexpression. $n = 8$, $\chi 2$ test. Data are shown as mean ± SD. **, $P < 0.01$; ***, $P < 0.001$; ****, $P < 0.0001$.

Supplementary Figure 3

A, The protein levels of IL-1α, IL-6, and IL-8 measured by ELISA in *LHX1*-knockout cells following treatment with SASP inhibitors. **B,** RT-qPCR analysis of *IL1A, IL6, IL8,*

and *CXCL10* following LHX1 overexpression. n = 3, unpaired t test. **C,** Immunoblotting of pRb, p21, and p16 following LHX1 overexpression. **D,** Colony formation assay in *LHX1*-knockout cells following treatment with SASP inhibitors. **E,** Quantification of the colony numbers in *LHX1*-knockout cells following treatment with SASP inhibitors. n = 3, one-way ANOVA. **F,** RT-qPCR analysis of *SOX2, POU5F1,* and *NANOG* in *LHX1*-knockout cells following treatment with SASP inhibitors. n = 3, one-way ANOVA. Data are shown as mean ± SD. *, $P < 0.05$; **, $P < 0.01$; ***, $P < 0.001$; ns, not significant.

Supplementary Figure 4

A, Colony formation assay in recipient cells following treatment with CM from *LHX1*-knockout cells. **B,** Quantification of the colony numbers. n = 3, unpaired t test. **C,** Sphere formation assay in recipient cells following treatment with conditioned medium from *LHX1*-knockout cells. Scale bar, 100 μm. **D,** Quantification of the sphere numbers in recipient cells. n = 3, unpaired t test. **E,** RT-qPCR analysis of *ALDHA1, SOX2, POU5F1,* and *NANOG* in recipient cells following treatment with CM from *LHX1*-knockout cells. n = 3, unpaired t test. **F,** Immunoblotting of pRb, p21, and p16 in *LHX1*-knockout cells following treatment with neutralizing-antibodies. **G,** Colony formation assay in *LHX1*-knockout cells following treatment with neutralizing-antibodies. **H,** Sphere formation assay in *LHX1*-knockout cells following treatment with neutralizing-antibodies. Scale bar, 100 μm. **I,** Quantification of the colony numbers. n = 3, one-way ANOVA. **J,** Quantification of the sphere numbers. n = 3, one-way ANOVA. Data are shown as mean ± SD. *, $P < 0.05$; **, $P < 0.01$; ***, $P < 0.001$; ****, $P < 0.0001$; ns,

not significant.

Supplementary Figure 5

A, The counts value of *cGAS* and *STING* following *LHX1* knockout. **B,** RT-qPCR analysis of *STING* in *LHX1* knockout FaDu cells. *n* = 3, one-way ANOVA. **C,** Immunoblotting analysis of STING, P-TBK1, TBK1, P-IRF3, and IRF3 in *LHX1* knockout FaDu cells. **D,** RT-qPCR analysis of *STING* following LHX1 overexpression. *n* = 3, unpaired *t* test. **E,** Immunoblotting analysis of STING following LHX1 overexpression. **F,** RT-qPCR analysis of *cGAS* following *LHX1* knockout. *n* = 3, one-way ANOVA. **G,** RT-qPCR analysis of *cGAS* following LHX1 overexpression. *n* = 3, unpaired *t* test. **H,** ELISA analysis of cGAMP in normal epithelial cells (HIOEC and HaCaT) and HNSCC cells (CAL27 and FaDu). **I,** ELISA analysis of cGAMP following *LHX1* knockout. **J,** ELISA analysis of cGAMP following LHX1 overexpression. **K,** Immunoblotting analysis of STING following re-expression of LHX1-WT, LHX1-ΔLIM, and LHX1-ΔHOX in *LHX1* knockout FaDu cells. **L,** RT-qPCR analysis of *STING* following re-expression of LHX1-WT, LHX1-ΔLIM, and LHX1-ΔHOX in *LHX1* knockout FaDu cells. *n* = 3, one-way ANOVA. **M,** ChIP-PCR analysis of LHX1 binding to different regions of the *STING* promoter in FaDu cells. **N,** ChIP-qPCR analysis of LHX1 binding to different regions of the *STING* promoter in FaDu cells. *n* = 3, unpaired *t* test. Data are shown as mean ± SD. *, $P < 0.05$; **, $P < 0.01$; ***, $P < 0.001$; ****, $P < 0.0001$; ns, not significant.

Supplementary Figure 6

A, Expression of *STING* in HNSCC and normal tissues from the TCGA database. n = 518 for tumors, n = 44 for normal tissues, unpaired t test. **B,** Promoter methylation level of *STING* in HNSCC and normal tissues from the TCGA database. n = 50 for normal tissues, n = 528 for tumors, unpaired t test. **C,** ChIP-qPCR analysis of H3K9me3 and H3K27me3 at the *STING* promoter following *LHX1*-knockout. n = 3, unpaired t test. **D,** Immunoblotting of pRb, p21, and p16 in *LHX1*-knockout cells following treatment with cGAMP. **E,** Colony formation assay in *LHX1*-knockout cells following treatment with cGAMP. F, Quantification of the colony numbers. n = 3, one-way ANOVA. Data are shown as mean ± SD. ***, $P < 0.001$; ns, not significant.

Supplementary Figure 7

A, The amino acid sequences of the LIM1 and LIM2 domains, as well as the sequences of the designed TAT, TAT-AE, and TAT-VM peptides. **B,** RT-qPCR analysis of *STING* in FaDu cells following treatment with different concentrations of TAT. **C,** RT-qPCR analysis of *STING* expression in FaDu cells when treated with different concentrations of TAT-AE and TAT-VM peptides. **D,** Co-IP results of HA-LDB1 and FLAG-LHX1 in 293T cells when treated with TAT-AE and TAT-VM peptides. **E,** RT-qPCR analysis of *STING* in HNSCC cells when treated with TAT-AE or TAT-VM peptides. n = 3, unpaired t test. **F,** Immunoblotting analysis of STING in HNSCC cells treated with TAT-AE and TAT-VM peptides. **G,** Colony formation assay following treatment with TAT-AE-DRI and TAT-VM-DRI peptides. **H,** Quantification of the colony numbers. n = 3,

one-way ANOVA. **I,** Effect of TAT-AE-DRI and TAT-VM-DRI peptides on the growth of CAL27-derived subcutaneous xenografts. $n = 8$, unpaired t test. **J,** Impact of TAT-AE-DRI and TAT-VM-DRI peptides on the survival of mice in the MOC2 orthotopic tongue tumor model. $n = 8$, Kaplan-Meier curve. Data are shown as mean ± SD. *** $P < 0.001$; **** $P < 0.0001$.

Supplementary Figure 8

A, Immunoblotting analysis of STING following LDB1 overexpression. **B,** RT-qPCR analysis of *STING* following LDB1 overexpression. $n = 3$, unpaired t test. **C,** Immunoblotting analysis of STING following *LDB1*-knockout. **D,** RT-qPCR analysis of *STING* following *LDB1*-knockout. $n = 3$, one-way ANOVA. **E,** Co-IP results of FLAG-LHX1 and HA-LDB1 in 293T cells, with FLAG-LHX1 serving as the bait protein. **F,** Co-IP results of HA-LDB1 and FLAG-LHX1 in 293T cells, with HA-LDB1 serving as the bait protein. **G,** Sequence diagram of the LDB1-WT and LDB1-ΔLID mutant. **H,** RT-qPCR analysis of *STING* following re-expression of LDB1-WT and LDB1-ΔLID mutant in *LDB1*-knockout FaDu cells. $n = 3$, one-way ANOVA. **I,** Immunoblotting analysis of STING following re-expression of LDB1-WT and LDB1-ΔLID mutant in *LDB1*-knockout FaDu cells. **J,** ChIP-PCR analysis of the binding of LHX1-WT and LHX1-3m mutant to the site1 region of *STING* promoter in FaDu cells. **K,** ChIP-qPCR analysis of the binding of LHX1-WT and LHX1-3m mutant to the site1 region of *STING* promoter in FaDu cells. $n = 3$, one-way ANOVA. **L,** RT-qPCR analysis of *STING* following re-expression of LHX1-WT or LHX1-3m mutant in *LHX1*

knockout FaDu cells. $n = 3$, one-way ANOVA. **M,** Immunoblotting analysis of STING following re-expression of LHX1-WT or LHX1-3m mutant in *LHX1* knockout FaDu cells. Data are shown as mean ± SD. **, $P < 0.01$; ***, $P < 0.001$; ****, $P < 0.0001$; ns, not significant.

Supplementary Figure 9

A, The amino acid sequences of the LIM1 and LIM2 domains, as well as the sequences of the designed TAT, TAT-AE, and TAT-VM peptides. **B,** Fluorescence microscopy images of FaDu with peptides conjugated to GFP protein. Scale bar, 50 μm. **C,** RT-qPCR analysis of *STING* in FaDu cells following treatment with different concentrations of TAT. **D,** RT-qPCR analysis of *STING* expression in FaDu cells when treated with different concentrations of TAT-AE and TAT-VM peptides. **E,** Co-IP results of HA-LDB1 and FLAG-LHX1 in 293T cells when treated with TAT-AE and TAT-VM peptides. **F,** RT-qPCR analysis of *STING* in HNSCC cells when treated with TAT-AE or TAT-VM peptides. $n = 3$, unpaired t test. **G,** Immunoblotting analysis of STING in HNSCC cells treated with TAT-AE and TAT-VM peptides. **H,** Co-IP results of LDB1 and LHX1, LMO1, LMO2 in FaDu cells when treated with TAT-AE-DRI and TAT-VM-DRI peptides. **I,** Colony formation assay following treatment with TAT-AE-DRI and TAT-VM-DRI peptides. **J,** Quantification of the colony numbers. $n = 3$, one-way ANOVA. $n = 8$, Kaplan-Meier curve. Data are shown as mean ± SD. *** $P < 0.001$; **** $P < 0.0001$.

Supplementary Figure 10

A, Effect of TAT-AE-DRI and TAT-VM-DRI peptides on the growth of CAL27-derived subcutaneous xenografts. *n* = 8, unpaired *t* test. **B,** Impact of TAT-AE-DRI and TAT-VM-DRI peptides on the survival of mice in the MOC2 orthotopic tongue tumor model. *n* = 8, Kaplan-Meier curve. **C,** IHC staining of Ki67, Cleaved Caspase-3, STING, p-TBK1, and p-IRF3 in tumor tissues from mice in the MOC1 orthotopic tongue tumor model. Scale bar, 50 μm. **D,** Statistical analysis of relative repression of Ki67, Cleaved Caspase-3, STING, p-TBK1, and p-IRF3. *n* = 8, unpaired *t* test. Data are shown as mean ± SD. **** $P < 0.0001$; ns, not significant.

Figure S1

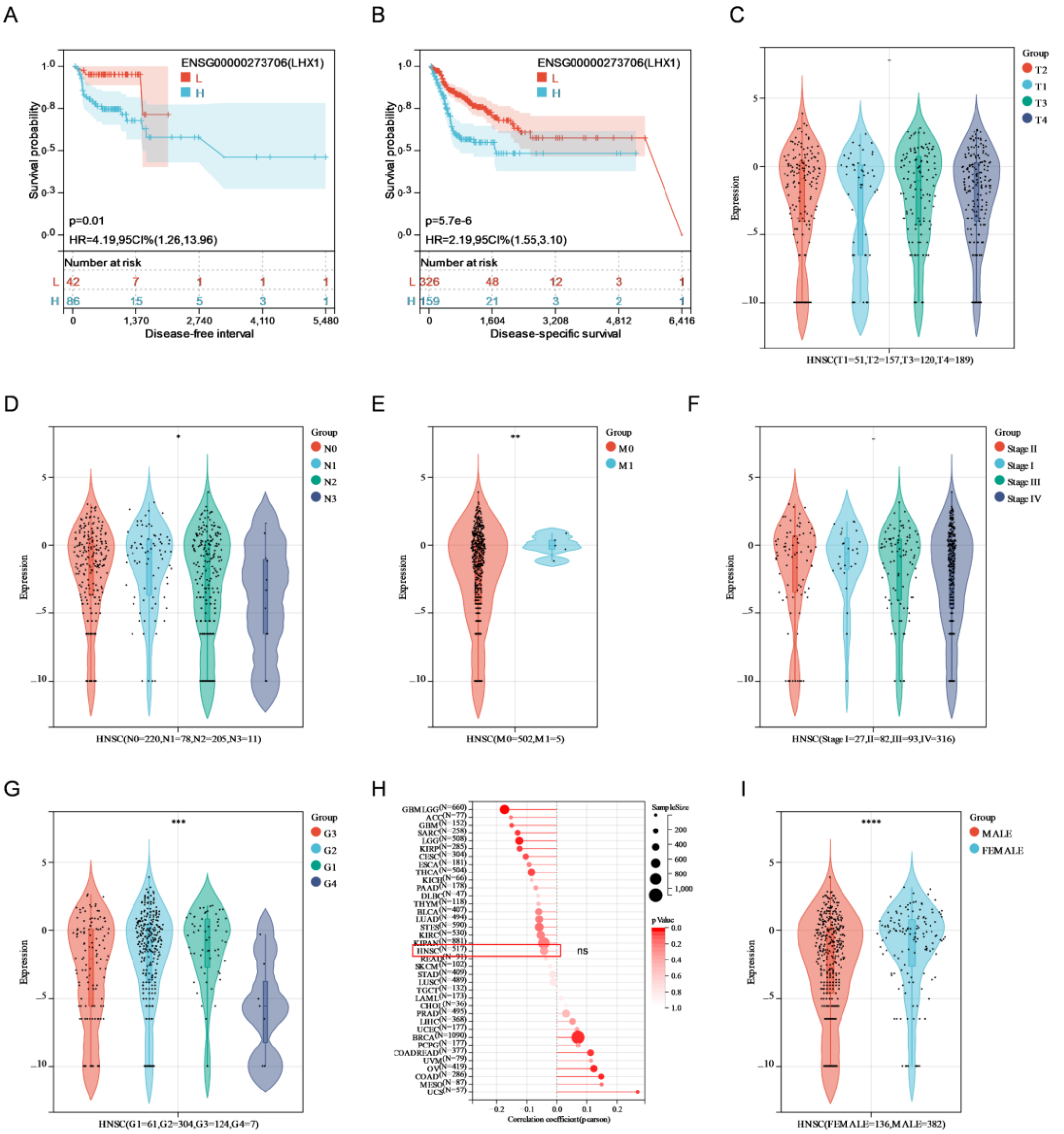
A
ENSG00000273706(LHX1)
L
H
Survival probability
p=0.01
HR=4.19,95CI%(1.26,13.96)
Number at risk
L 42 7 1 1 1
H 86 15 5 3 1
0 1,370 2,740 4,110 5,480
Disease-free interval
B
ENSG00000273706(LHX1)
L
H
Survival probability
p=5.7e-6
HR=2.19,95CI%(1.55,3.10)
Number at risk
L 326 48 12 3 1
H 159 21 3 2 1
0 1,604 3,208 4,812 6,416
Disease-specific survival
C
Group
T2
T1
T3
T4
Expression
HNSC(T1=51,T2=157,T3=120,T4=189)
D
Group
N0
N1
N2
N3
Expression
HNSC(N0=220,N1=78,N2=205,N3=11)
E
Group
M0
M1
Expression
HNSC(M0=502,M1=5)
F
Group
Stage II
Stage I
Stage III
Stage IV
Expression
HNSC(Stage I=27,II=82,III=93,IV=316)
G
Group
G3
G2
G1
G4
Expression
HNSC(G1=61,G2=304,G3=124,G4=7)
H
ns
SampleSize
p Value
Correlation coefficient(pearson)
I
Group
MALE
FEMALE
Expression
HNSC(FEMALE=136,MALE=382)

Figure S2

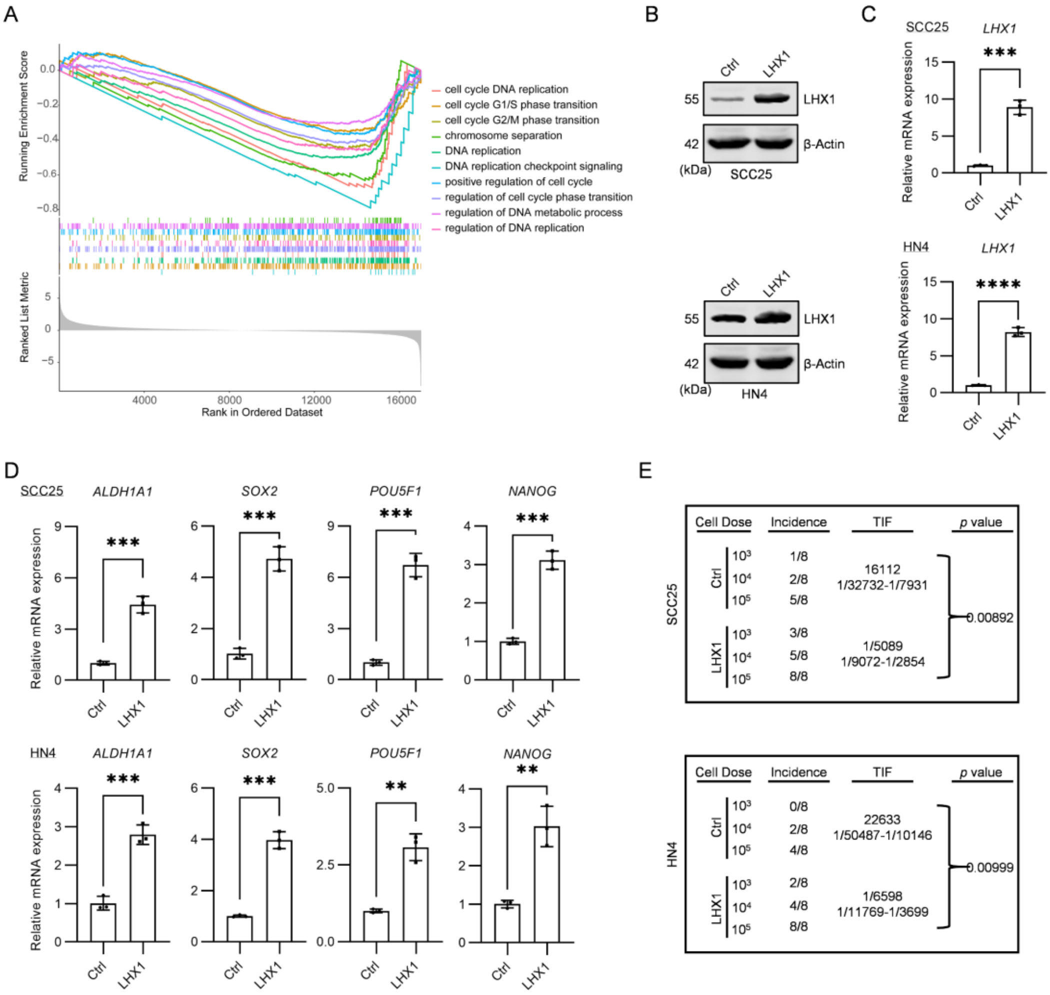
A
Running Enrichment Score
0.0
−0.2
−0.4
−0.6
−0.8
cell cycle DNA replication
cell cycle G1/S phase transition
cell cycle G2/M phase transition
chromosome separation
DNA replication
DNA replication checkpoint signaling
positive regulation of cell cycle
regulation of cell cycle phase transition
regulation of DNA metabolic process
regulation of DNA replication
Ranked List Metric
5
0
−5
4000
8000
12000
16000
Rank in Ordered Dataset
B
Ctrl
LHX1
55
LHX1
42
β-Actin
(kDa)
SCC25
Ctrl
LHX1
55
LHX1
42
β-Actin
(kDa)
HN4
C
SCC25
LHX1
Relative mRNA expression
15
10
5
0
***
Ctrl
LHX1
HN4
LHX1
Relative mRNA expression
15
10
5
0
****
Ctrl
LHX1
D
SCC25
ALDH1A1
SOX2
POU5F1
NANOG
Relative mRNA expression
***
***
***
***
Ctrl
LHX1
HN4
ALDH1A1
SOX2
POU5F1
NANOG
Relative mRNA expression
***
***
**
**
Ctrl
LHX1
E
SCC25
Cell Dose
Incidence
TIF
p value
Ctrl
10^3 1/8
10^4 2/8
10^5 5/8
16112
1/32732-1/7931
LHX1
10^3 3/8
10^4 5/8
10^5 8/8
1/5089
1/9072-1/2854
0.00892
HN4
Cell Dose
Incidence
TIF
p value
Ctrl
10^3 0/8
10^4 2/8
10^5 4/8
22633
1/50487-1/10146
LHX1
10^3 2/8
10^4 4/8
10^5 8/8
1/6598
1/11769-1/3699
0.00999

Figure S3

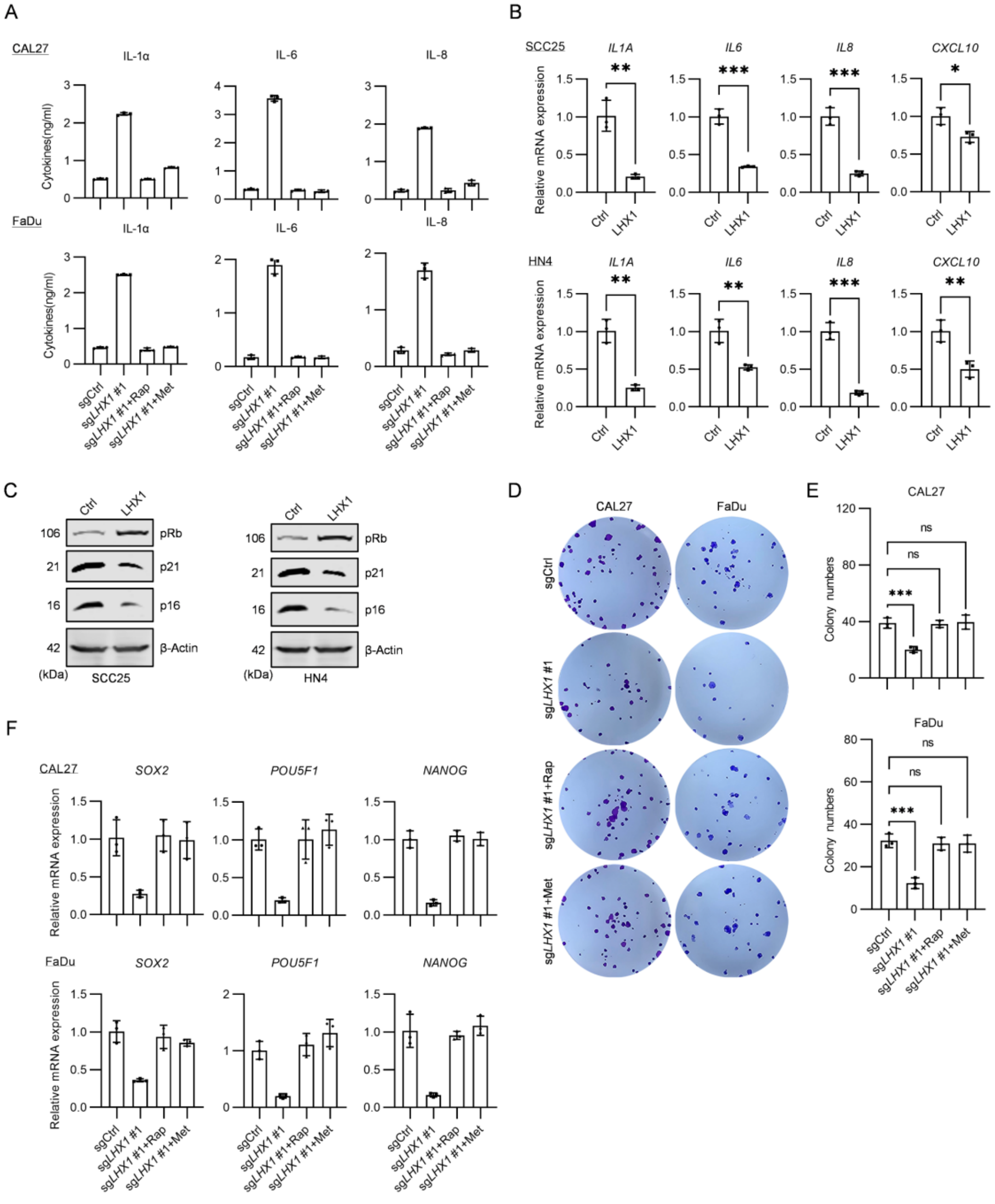

A
CAL27
IL-1α
IL-6
IL-8
Cytokines(ng/ml)
FaDu
sgCtrl
sgLHX1 #1
sgLHX1 #1+Rap
sgLHX1 #1+Met
B
SCC25
IL1A
IL6
IL8
CXCL10
Relative mRNA expression
Ctrl
LHX1
HN4
C
Ctrl
LHX1
106
pRb
21
p21
16
p16
42
β-Actin
(kDa)
SCC25
HN4
D
CAL27
FaDu
sgCtrl
sgLHX1 #1
sgLHX1 #1+Rap
sgLHX1 #1+Met
E
Colony numbers
ns
F
SOX2
POU5F1
NANOG

Figure S4

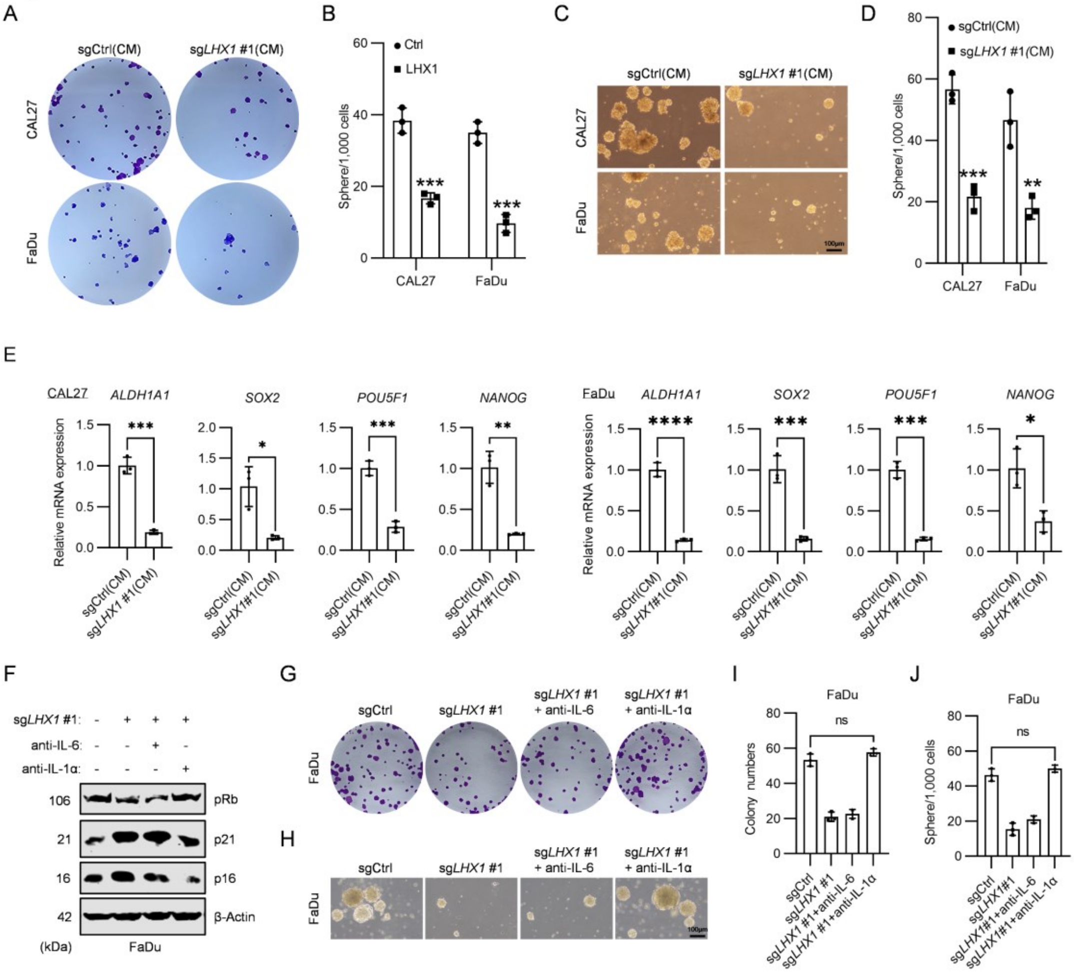
A
sgCtrl(CM)
sgLHX1 #1(CM)
CAL27
FaDu
B
Ctrl
LHX1
Sphere/1,000 cells
60
40
20
0
***
***
CAL27
FaDu
C
sgCtrl(CM)
sgLHX1 #1(CM)
CAL27
FaDu
100μm
D
sgCtrl(CM)
sgLHX1 #1(CM)
Sphere/1,000 cells
80
60
40
20
0
***
**
CAL27
FaDu
E
CAL27
ALDH1A1
SOX2
POU5F1
NANOG
Relative mRNA expression
***
*
***
**
sgCtrl(CM)
sgLHX1 #1(CM)
FaDu
ALDH1A1
SOX2
POU5F1
NANOG
Relative mRNA expression
****
***
***
*
sgCtrl(CM)
sgLHX1#1(CM)
F
sgLHX1 #1:
anti-IL-6:
anti-IL-1α:
106
pRb
21
p21
16
p16
42
β-Actin
(kDa)
FaDu
G
sgCtrl
sgLHX1 #1
sgLHX1 #1 + anti-IL-6
sgLHX1 #1 + anti-IL-1α
FaDu
H
sgCtrl
sgLHX1 #1
sgLHX1 #1 + anti-IL-6
sgLHX1 #1 + anti-IL-1α
FaDu
100μm
I
FaDu
ns
Colony numbers
80
60
40
20
0
sgCtrl
sgLHX1 #1
sgLHX1 #1+anti-IL-6
sgLHX1 #1+anti-IL-1α
J
FaDu
ns
Sphere/1,000 cells
80
60
40
20
0
sgCtrl
sgLHX1#1
sgLHX1#1+anti-IL-6
sgLHX1#1+anti-IL-1α

Figure S5

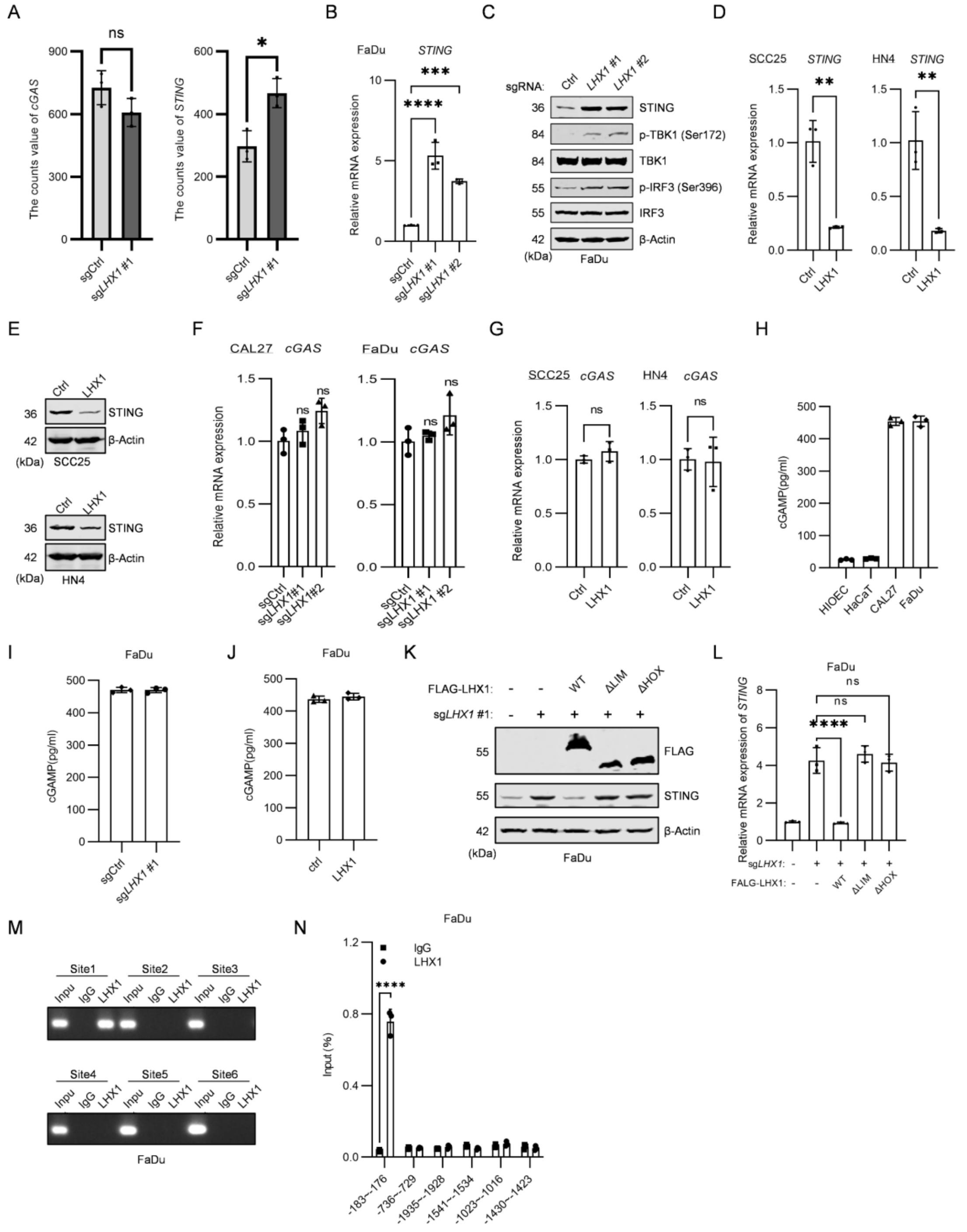
A
ns
*
The counts value of cGAS
The counts value of STING
sgCtrl
sgLHX1 #1
B
FaDu
STING
***
****
Relative mRNA expression
sgCtrl
sgLHX1 #1
sgLHX1 #2
C
sgRNA:
Ctrl
LHX1 #1
LHX1 #2
STING
p-TBK1 (Ser172)
TBK1
p-IRF3 (Ser396)
IRF3
β-Actin
(kDa)
FaDu
D
SCC25
STING
HN4
STING
**
Relative mRNA expression
Ctrl
LHX1
E
Ctrl
LHX1
STING
β-Actin
(kDa)
SCC25
HN4
F
CAL27 cGAS
FaDu cGAS
ns
Relative mRNA expression
sgCtrl
sgLHX1#1
sgLHX1#2
sgLHX1 #1
sgLHX1 #2
G
SCC25 cGAS
HN4 cGAS
ns
Relative mRNA expression
Ctrl
LHX1
H
cGAMP(pg/ml)
HIOEC
HaCaT
CAL27
FaDu
I
FaDu
cGAMP(pg/ml)
sgCtrl
sgLHX1 #1
J
FaDu
cGAMP(pg/ml)
ctrl
LHX1
K
FLAG-LHX1:
WT
ΔLIM
ΔHOX
sgLHX1 #1:
FLAG
STING
β-Actin
(kDa)
FaDu
L
FaDu
ns
****
Relative mRNA expression of STING
sgLHX1:
FALG-LHX1:
WT
ΔLIM
ΔHOX
M
Site1
Site2
Site3
Site4
Site5
Site6
Inpu
IgG
LHX1
FaDu
N
FaDu
IgG
LHX1
****
Input (%)
-183~-176
-736~-729
-1935~-1928
-1541~-1534
-1023~-1016
-1430~-1423

Figure S6

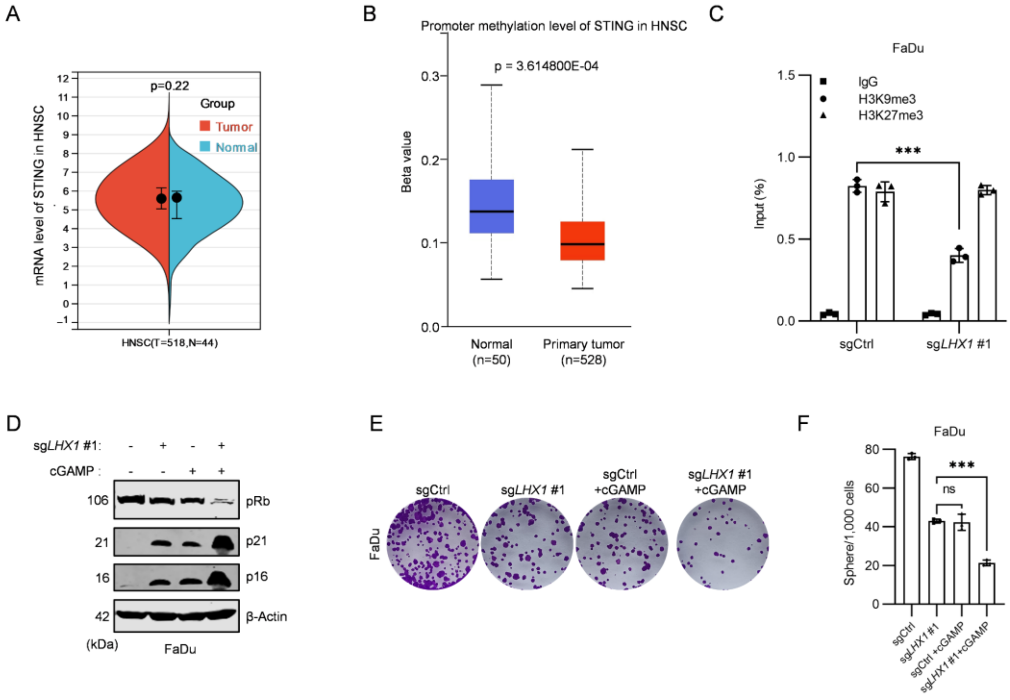

A
p=0.22
Group
Tumor
Normal
mRNA level of STING in HNSC
HNSC(T=518,N=44)
B
Promoter methylation level of STING in HNSC
p = 3.614800E-04
Beta value
Normal
(n=50)
Primary tumor
(n=528)
C
FaDu
IgG
H3K9me3
H3K27me3
Input (%)
***
sgCtrl
sgLHX1 #1
D
sgLHX1 #1:
cGAMP :
pRb
p21
p16
β-Actin
(kDa)
FaDu
E
sgCtrl
sgLHX1 #1
sgCtrl
+cGAMP
sgLHX1 #1
+cGAMP
FaDu
F
FaDu
Sphere/1,000 cells
ns
***
sgCtrl
sgLHX1 #1
sgCtrl+cGAMP
sgLHX1#1+cGAMP

Figure S7

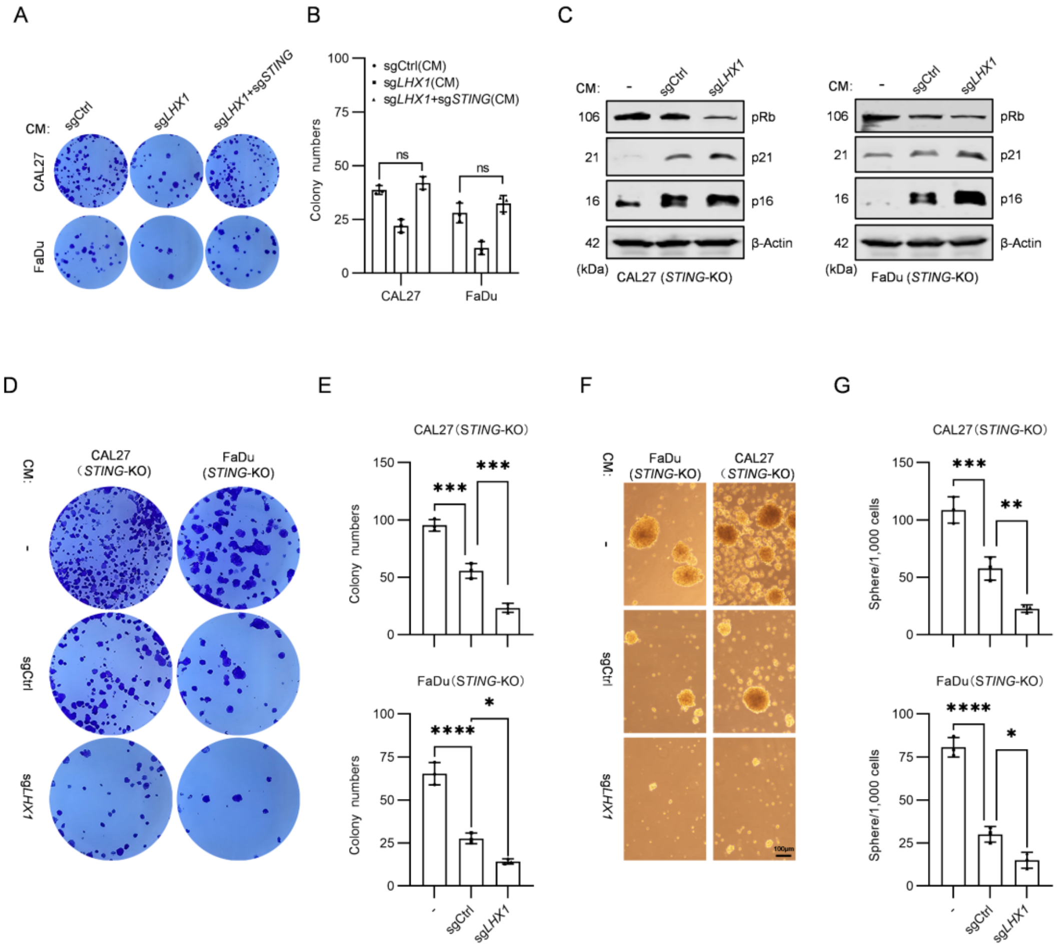

A
CM:
sgCtrl
sgLHX1
sgLHX1+sgSTING
CAL27
FaDu
B
sgCtrl(CM)
sgLHX1(CM)
sgLHX1+sgSTING(CM)
Colony numbers
ns
ns
CAL27
FaDu
C
CM:
-
sgCtrl
sgLHX1
106
21
16
42
(kDa)
pRb
p21
p16
β-Actin
CAL27 (STING-KO)
FaDu (STING-KO)
D
CM:
CAL27 (STING-KO)
FaDu (STING-KO)
-
sgCtrl
sgLHX1
E
CAL27(STING-KO)
FaDu(STING-KO)
Colony numbers
F
CM:
FaDu (STING-KO)
CAL27 (STING-KO)
100μm
G
CAL27(STING-KO)
FaDu(STING-KO)
Sphere/1,000 cells

Figure S8

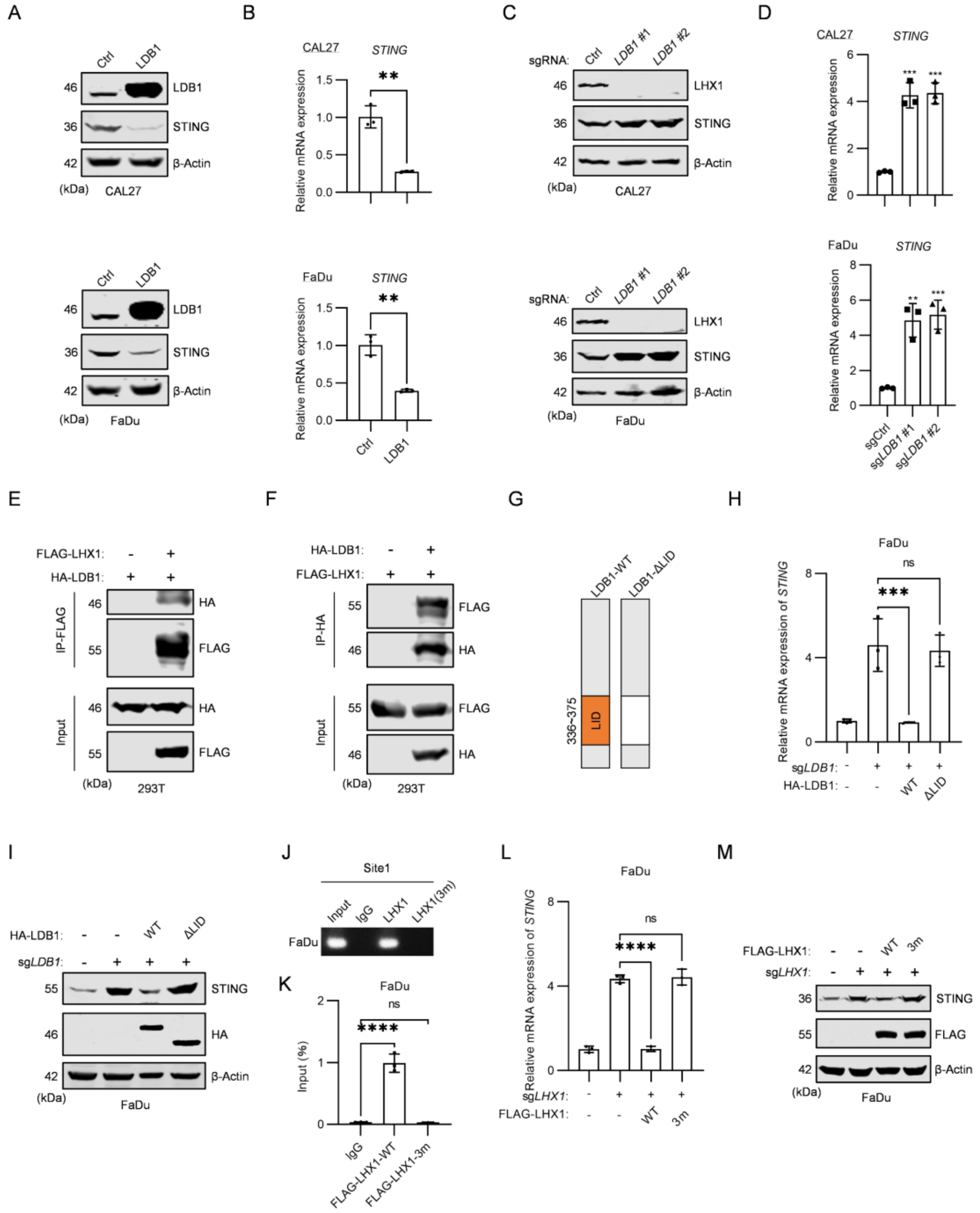

A
Ctrl
LDB1
46
LDB1
36
STING
42
β-Actin
(kDa)
CAL27
FaDu
B
CAL27
STING
Relative mRNA expression
FaDu
Ctrl
LDB1
C
sgRNA:
Ctrl
LDB1 #1
LDB1 #2
LHX1
STING
β-Actin
CAL27
FaDu
D
CAL27
STING
Relative mRNA expression
FaDu
sgCtrl
sgLDB1 #1
sgLDB1 #2
E
FLAG-LHX1:
HA-LDB1:
IP-FLAG
Input
HA
FLAG
293T
F
HA-LDB1:
FLAG-LHX1:
IP-HA
Input
FLAG
HA
293T
G
LDB1-WT
LDB1-ΔLID
336-375
LID
H
FaDu
ns
Relative mRNA expression of STING
sgLDB1:
HA-LDB1:
WT
ΔLID
I
HA-LDB1:
sgLDB1:
WT
ΔLID
STING
HA
β-Actin
FaDu
J
Site1
Input
IgG
LHX1
LHX1(3m)
FaDu
K
FaDu
ns
Input (%)
IgG
FLAG-LHX1-WT
FLAG-LHX1-3m
L
FaDu
ns
Relative mRNA expression of STING
sgLHX1:
FLAG-LHX1:
WT
3m
M
FLAG-LHX1:
sgLHX1:
WT
3m
STING
FLAG
β-Actin
FaDu

Figure S9

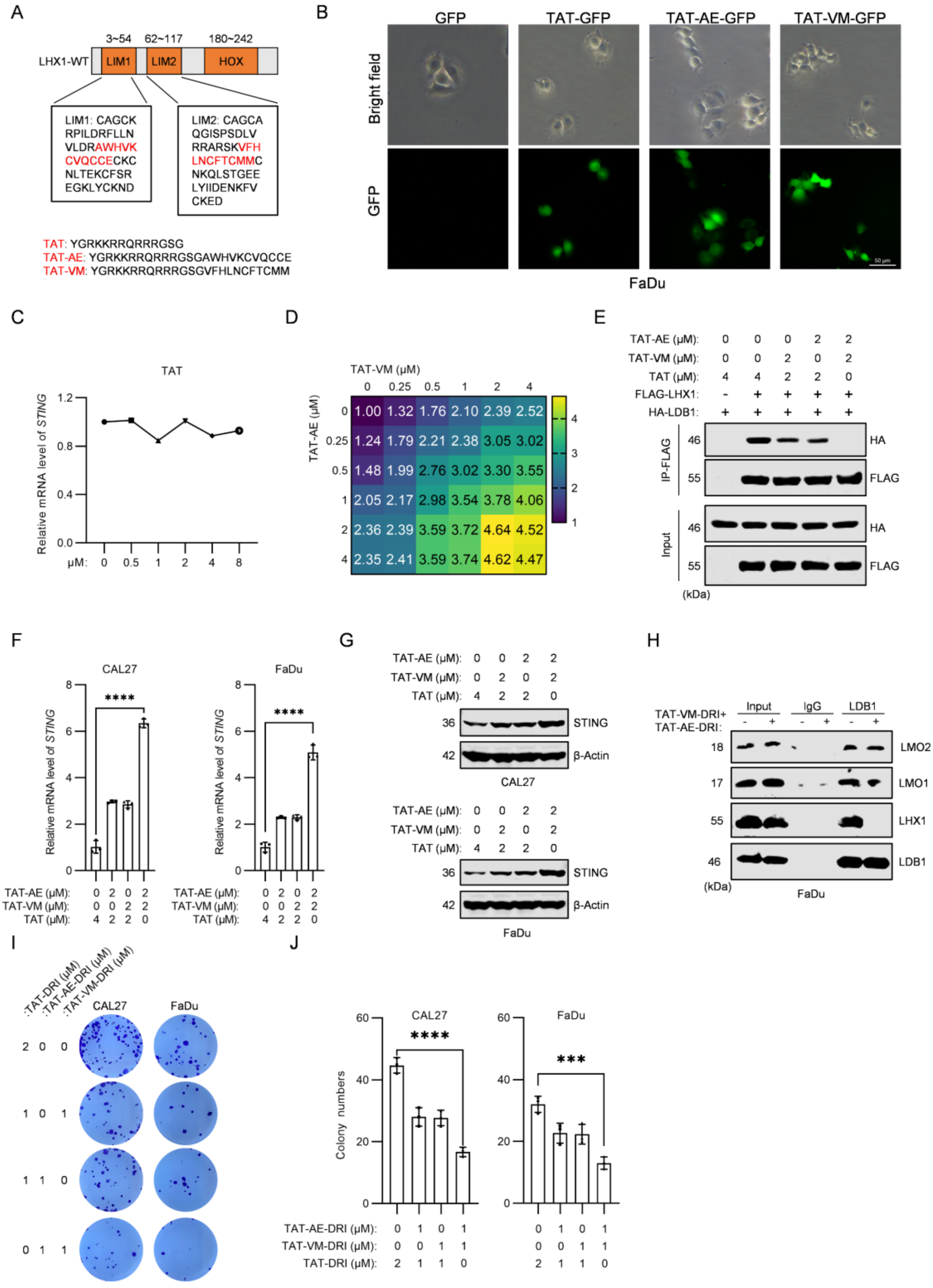
A
3~54
62~117
180~242
LHX1-WT
LIM1
LIM2
HOX
LIM1: CAGCK RPILDRFLLN VLDRAWHVK CVQCCECKC NLTEKCFSR EGKLYCKND
LIM2: CAGCA QGISPSDLV RRARSKVFH LNCFTCMMC NKQLSTGEE LYIIDENKFV CKED
TAT: YGRKKRRQRRRGSG
TAT-AE: YGRKKRRQRRRGSGAWHVKCVQCCE
TAT-VM: YGRKKRRQRRRGSGVFHLNCFTCMM
B
GFP
TAT-GFP
TAT-AE-GFP
TAT-VM-GFP
Bright field
GFP
FaDu
C
TAT
Relative mRNA level of STING
μM: 0 0.5 1 2 4 8
D
TAT-VM (μM)
TAT-AE (μM)
E
TAT-AE (μM): 0 0 0 2 2
TAT-VM (μM): 0 0 2 0 2
TAT (μM): 4 4 2 2 0
FLAG-LHX1: - + + + +
HA-LDB1: + + + + +
IP-FLAG
Input
HA
FLAG
(kDa)
F
CAL27
FaDu
****
TAT-AE (μM): 0 2 0 2
TAT-VM (μM): 0 0 2 2
TAT (μM): 4 2 2 0
G
TAT-AE (μM): 0 0 2 2
TAT-VM (μM): 0 2 0 2
TAT (μM): 4 2 2 0
STING
β-Actin
CAL27
FaDu
H
TAT-VM-DRI+
TAT-AE-DRI:
Input
IgG
LDB1
LMO2
LMO1
LHX1
LDB1
(kDa)
FaDu
I
TAT-DRI (μM)
TAT-AE-DRI (μM)
TAT-VM-DRI (μM)
CAL27
FaDu
J
Colony numbers
****
***
TAT-AE-DRI (μM): 0 1 0 1
TAT-VM-DRI (μM): 0 0 1 1
TAT-DRI (μM): 2 1 1 0

Figure S10

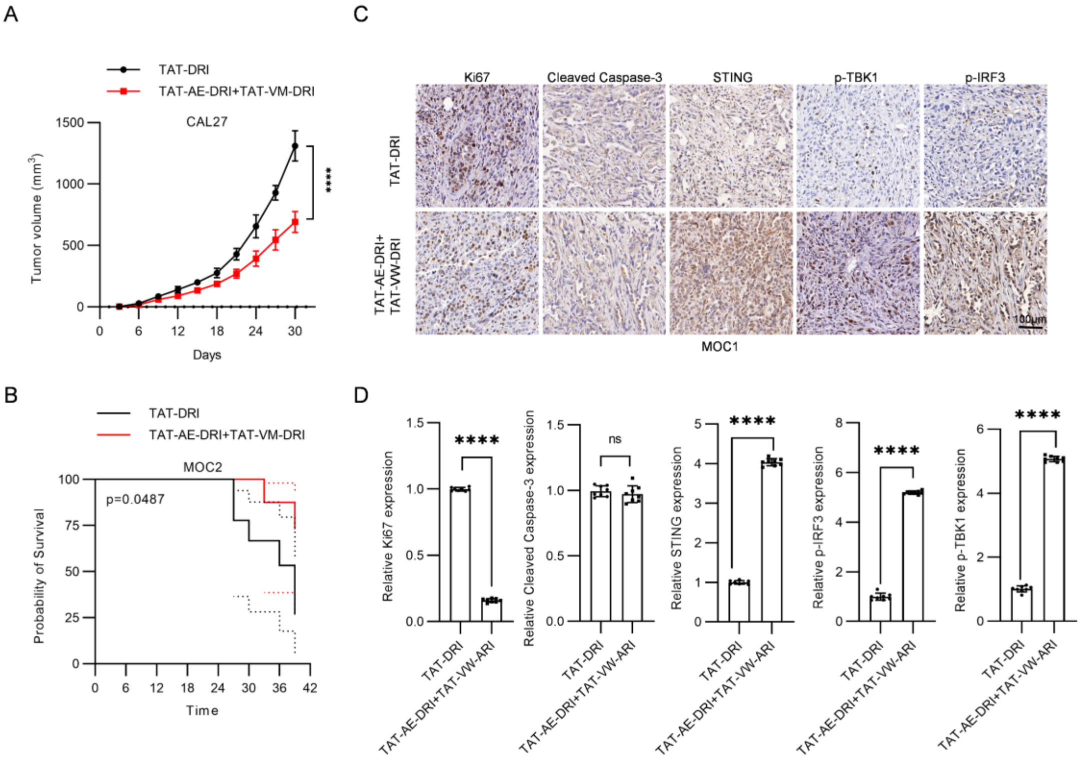

A
TAT-DRI
TAT-AE-DRI+TAT-VM-DRI
CAL27
Tumor volume (mm3)
Days
****
B
TAT-DRI
TAT-AE-DRI+TAT-VM-DRI
MOC2
p=0.0487
Probability of Survival
Time
C
Ki67
Cleaved Caspase-3
STING
p-TBK1
p-IRF3
TAT-DRI
TAT-AE-DRI+ TAT-VW-DRI
100μm
MOC1
D
Relative Ki67 expression
Relative Cleaved Caspase-3 expression
Relative STING expression
Relative p-IRF3 expression
Relative p-TBK1 expression
ns
****
TAT-DRI
TAT-AE-DRI+TAT-VW-ARI

Table S1. Detailed information of patients.

Patients	Age	Location	TNM	Relapse	Grade
1	53	Mouth Floor	T3N0M0	NO	II-III
2	55	Buccal Mucosa	T2N1M0	NO	II
3	67	Buccal Mucosa	T2N0M0	NO	I-II
4	46	Tongue	T2N1M0	YES	III
5	39	Tongue	T3N1M0	NO	III
6	72	Gingiva	T2N0M0	NO	II
7	73	Buccal Mucosa	T1N0M0	NO	I-II
8	71	Gingiva	T2N0M0	YES	II-III
9	71	Tongue	T3N0M0	YES	III
10	48	Tongue	T1N0M0	NO	I-II
11	62	Buccal Mucosa	T2N0M0	NO	II
12	57	Buccal Mucosa	T2N0M0	NO	II
13	37	Tongue	T3N1M0	NO	III
14	32	Tongue	T3N0M0	YES	II-III
15	75	Tongue	T2N0M0	NO	II
16	34	Tongue	T2N0M0	NO	II
17	48	Tongue	T1N0M0	NO	I
18	69	Soft Palate	T2N1M0	NO	III
19	49	Tongue	T1N2M0	NO	IV
20	44	Buccal Mucosa	T3N1M0	NO	III
21	36	Tongue	T3N0M0	YES	II-III

Table S2. The primers for sgRNAs.

Genes	Species	Primers
Ctrl	Human	F: CACCGACGGAGGCTAAGCGTCGCAA
		R: AAACTTGCGACGCTTAGCCTCCGTC
LHX1 #1	Human	F: CACCGTCTCCGCACCAGGTCGCTAG
		R: AAACCTAGCGACCTGGTGCGGAGAC
LHX1 #2	Human	F: CACCGCGGCTGCAAAAGGCCCATCC
		R: AAACGGATGGGCCTTTTGCAGCCGC
STING	Human	F: CACCGCTGGGACTGCTGTTAAACG
		R: AAACCGTTTAACAGCAGTCCCAGC
LDB1 #1	Human	F: CACCGGAAAGGCGTTGCCGTTCGG
		R: AAACCCGAACGGCAACGCCTTTCC
LDB1 #2	Human	F: CACCGGAGTGTGACAATCTCTGGT
		R: AAACACCAGAGATTGTCACACTCC

Table S3. The primers used for RT-qPCR.

Genes	Species	Primers
ACTB	Human	F: CCTGGCACCCAGCACAAT
		R: GGGCCGGACTCGTCATAC
LHX1	Human	F: GCCCACCCGCCACATCC
		R: TGCTTCATCCTCCGCTCCTTG
ALDH1A1	Human	F: CCGTGGCGTACTATGGATGC
		R: GCAGCAGACGATCTCTTTCGAT
SOX2	Human	F: GCCGAGTGGAAACTTTTGTCG
		R: GGCAGCGTGTACTTATCCTTCT
POU5F1	Human	F: CTGGGTTGATCCTCGGACCT
		R: CCATCGGAGTTGCTCTCCA
NANOG	Human	F: CCCCAGCCTTTACTCTTCCTA
		R: CCAGGTTGAATTGTTCCAGGTC
IL1A	Human	F: TGGTAGTAGCAACCAACGGGA
		R: ACTTTGATTGAGGGCGTCATTC
IL6	Human	F: ACTCACCTCTTCAGAACGAATTG
		R: CCATCTTTGGAAGGTTCAGGTTG
IL8	Human	F: TTTTGCCAAGGAGTGCTAAAGA
		R: AACCCTCTGCACCCAGTTTTC
CXCL10	Human	F: CACCATGAATCAAACTGCGA
		R: GCTGATGCAGGTACAGCGT
STING1	Human	F: CTAGGAGAGCCACCAGAGCAC
		R: AGAAATAGATGGACAGCAGCAACAG
cGAS	Human	F: AAGAAGAAACATGGCGGCTATCC
		R: AAGGCTGAGGCAGGAGAATGG

Table S4. The primers used in the ChIP assay.

Binding sites	Binding sequence	Primers
Site1	GCAATTAC	F: CGGCCTCCCAAAGCGCTGGG
		R: GGTTTCTGCCCAGAAGGCGG
Site2	TCAATTAC	F: CAGAGCAAGCTGGGCTTTGG
		R: TTTTATAGATGGATAATCCG
Site3	CCAATTAT	F: GTCTATTTTTACGTTTAGGT
		R: GGCTGGGTTTCCCCACTCAT
Site4	GCCATCAC	F: AAGCAGTTCTCCTGCCTCAG
		R: CAACATGGAGAAACCCCATC
Site5	GCCATCAC	F: TTTTTTTGAGATAGGGTATC
		R: CTGAGGTGGGAGCATCACTT
Site6	GCAATTTG	F: AGGATGGTCTTGAACTCCTG
		R: CGGCAACAAGAGTCAAACTC
